# Microbial Extracellular Polymeric Substances (EPSs) in Ocean Systems

**DOI:** 10.3389/fmicb.2017.00922

**Published:** 2017-05-26

**Authors:** Alan W. Decho, Tony Gutierrez

**Affiliations:** ^1^Department of Environmental Health Sciences, Arnold School of Public Health, University of South Carolina, ColumbiaSC, United States; ^2^School of Engineering and Physical Sciences, Heriot-Watt UniversityEdinburgh, United Kingdom

**Keywords:** EPS, biofilm, organic matter, oceans research, bacteria

## Abstract

Microbial cells (i.e., bacteria, archaea, microeukaryotes) in oceans secrete a diverse array of large molecules, collectively called extracellular polymeric substances (EPSs) or simply *exopolymers*. These secretions facilitate attachment to surfaces that lead to the formation of structured ‘*biofilm*’ communities. In open-water environments, they also lead to formation of organic colloids, and larger aggregations of cells, called ‘*marine snow.’* Secretion of EPS is now recognized as a fundamental microbial adaptation, occurring under many environmental conditions, and one that influences many ocean processes. This relatively recent realization has revolutionized our understanding of microbial impacts on ocean systems. EPS occur in a range of molecular sizes, conformations and physical/chemical properties, and polysaccharides, proteins, lipids, and even nucleic acids are actively secreted components. Interestingly, however, the physical ultrastructure of how individual EPS interact with each other is poorly understood. Together, the EPS matrix molecules form a three-dimensional architecture from which cells may localize extracellular activities and conduct cooperative/antagonistic interactions that cannot be accomplished efficiently by free-living cells. EPS alter optical signatures of sediments and seawater, and are involved in biogeomineral precipitation and the construction of microbial macrostructures, and horizontal-transfers of genetic information. In the water-column, they contribute to the formation of marine snow, transparent exopolymer particles (TEPs), sea-surface microlayer biofilm, and marine oil snow. Excessive production of EPS occurs during later-stages of phytoplankton blooms as an excess metabolic by product and releases a carbon pool that transitions among dissolved-, colloidal-, and gel-states. Some EPS are highly labile carbon forms, while other forms appear quite refractory to degradation. Emerging studies suggest that EPS contribute to efficient trophic-transfer of environmental contaminants, and may provide a protective refugia for pathogenic cells within marine systems; one that enhances their survival/persistence. Finally, these secretions are prominent in ‘extreme’ environments ranging from sea-ice communities to hypersaline systems to the high-temperatures/pressures of hydrothermal-vent systems. This overview summarizes some of the roles of exopolymer in oceans.

## Overview

**FIGURE 1:**
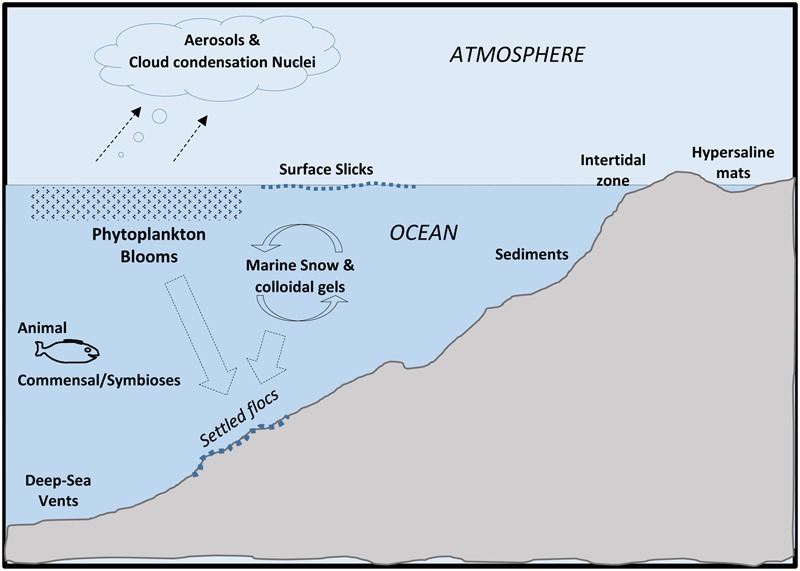
**Major locations of extracellular polymeric substances (EPSs) in Oceans**.

Microorganisms (e.g., bacteria, archaea, microeukaryotes) reside in ocean systems in an assortment of physical states ranging from free-living cells to complex communities attached to surfaces and to each other (Moran, 2015; Brussaard et al., 2016). Over the span of different ocean environments, microbial flora take up dissolved organics and ions, and then secrete polymeric organic compounds. These secretions, called *exopolymers* or extracellular polymeric substances (EPSs), are abundant and become mixed with other forms of organic matter within ocean systems. It was recognized early on, that under the fluctuating, and often less-predictable conditions of natural systems (compared to those of a laboratory culture flask), the attachment of microbes to surfaces, or to each other, offers a degree of environmental stability not experienced by free-living (non-attached) cells (ZoBell and Allen, 1935). An initial understanding of the purposeful secretion of EPS and their potential stabilizing effects for microbial cells initially emerged during the last century. It is now realized and mostly accepted that many bacteria and other microorganisms occur in a biofilm state; either attached to surfaces or as suspended-aggregates in the water column. EPS, the subject of this overview, consist of a wide range of molecules and provide selective adaptations for the cells that produce them, which in turn, influence broader ocean processes (**Figure 1**).

## EPS: A Microbial Adaptation for Aggregation and Attachment

Extracellular polymeric substance are purposefully produced by microbes: (a) as secretions of biofilms that secure attachment and enhance their local environment, and/or (b) as metabolic-excess waste products. The differences between these two processes is easily discernable but becomes important when addressing the provenance of organic matter and the roles that EPS contribute to ocean systems. *It is important to point out that EPS are not an essential component to microbial life* (i.e., *cells can survive and grow without them), but rather their secretion strongly enhances the survival, metabolic efficiency and adaptation of cells.*

### The Biofilm State

The term ‘*biofilm*’ was coined long ago (Costerton et al., 1987), and refers to microbial cells that have attached to a surface or aggregated with each other, and have secreted a gelatinous matrix of EPS. The ability of a microbial cell, such as a bacterium, to attach, secrete EPS and form a biofilm under laboratory conditions, is well-established. The secretion of EPS (by cells) is a key emergent property of the biofilm (Flemming and Wingender, 2010; Flemming et al., 2016); the property that directly influences adaptations that cells utilize to enhance their efficiency and survival. The secretion of an EPS matrix represents, in the broadest sense, an extension of the cell. The presence of EPS facilitates the self-organization of cells into localized communities, and provides biofilm cells with an enhanced capability for: trapping other organics and localizing their digestion by extracellular enzymes, coordinating cell–cell chemical communication [quorum sensing (QS)], facilitating gene-exchange, and provides a degree of physical stability. The EPS often form a localization matrix for other molecules, keeping them in spatial proximity to cells where they can be efficiently utilized.

It is now generally recognized within microbiology that the ‘biofilm state’ is an omnipresent feature of microbial flora in most environments (Hall-Stoodley et al., 2004). Biofilms occur under a wide range of conditions and environments, and whose influences span aquatic, terrestrial, the epi- and endo-biont communities of plants and animals, which can be commensal, symbiotic or pathogenic. The cells within a biofilm can move, and periodically reorient themselves in relation to one another, and in doing so can resist invasion by other cells (Houry et al., 2012). The EPS matrix of biofilms provides a three-dimensional architecture framework that allows the arrangements of cells movements relative to other microbes as well as positioning among sharp geochemical gradients (Decho, 2000b). This will not be discussed further here, but directly contributes to the remarkable plasticity of biofilm cells. The EPS form a matrix of largely anionic molecules near cells, affording them with a proximal environment that is more stabilizing, and conducive to manipulation by the cell (**Table 1**), and one that contributes to broader ocean processes. However, in this overview we will not discuss biofilms as systems, except with regard to their secretion of EPS.

**Table 1:** Major EPS physical/chemical properties and functions and influence(s) on ocean processes.

EPS property	Influence on Ocean process	Reference^∗^
**Physical state**		
Gel/solution state**:**	-Aggregation- formation of colloids, TEP, and marine snow; -Contribution to carbon flux;	-Alldredge et al., 1993; Verdugo, 1994; Passow, 2002; Simon et al., 2002; Verdugo and Santschi, 2010; -Long and Azam, 2001a; Engel et al., 2004; Wurl, 2009;
Amphiphilic:	-Dispersion of oil/ MOS and other hydrophobic contaminants; -Hydrophobic microdomains/contaminants; -Sea surface slicks and aerosols;	-Niu et al., 2011; Passow et al., 2012; Gutierrez et al., 2013; Valentine et al., 2014; Daly et al., 2016; -Decho, 2000b; Lawrence et al., 2007, 2016; -Kuznetsova et al., 2005; Facchini et al., 2008; Leck and Bigg, 2008; Wurl and Holmes, 2008; Fuentes et al., 2010
**Chemical composition**		
Degradability	-Consumer food source; -DOM/POM turnover/refractory OM pool;	-Decho and Moriarty, 1990; Decho and Lopez, 1993; Schlekat et al., 1998, 1999, 2000; Selck et al., 1999; -Ogawa et al., 2001; Benner, 2002; Decho et al., 2005; Repeta and Aluwihare, 2006; Walker et al., 2016;
Reactive groups	-Sorption of organic- /inorganic- ions; -Enhancement of iron bioavailability; -Biogeomineral precipitation;	-Bhaskar and Bhosle, 2006; Zhang et al., 2008; Braissant et al., 2009; Gutierrez et al., 2012; Deschatre et al., 2013; -Boyd et al., 2007; Hassler et al., 2011b; -Reid et al., 2000; Arp et al., 2001; Kawaguchi and Decho, 2002a; Dupraz et al., 2009; Obst et al., 2009
Excess metabolite	-Secretion by late-stage plankton blooms;	-Aluwihare et al., 1997; Bhaskar and Bhosle, 2005;
**Protection/enhancement of microbial activities**		
Diffusion-slowing/localization close to cells:	-*e*-Enzymes and hydrolysis products; -Quorum sensing signals; -Enhancement of microscale gradients; -Lipid vesicles and antibiotics;	-Smith et al., 1992; Stewart, 2002; Flemming and Wingender, 2010; Jatt et al., 2015; Sutherland, 2016; -Decho et al., 2009; Hmelo et al., 2011; Decho, 2015; - Visscher and Stolz, 2005; Vasconcelos et al., 2006; -Mashburn and Whiteley, 2005; Schooling et al., 2009; Biller et al., 2014;
Sorption/trapping:	-Concentration of viruses/phages; -Larval settlement cues;	- Drake et al., 2007; Dupuy et al., 2014; Guizien et al., 2014; -Holmström et al., 2002; Franks et al., 2006; Tran and Hadfield, 2011; Nielsen et al., 2015;
Stickiness/cohesiveness:	-Biofilm and microbial mat formation; -Sediment stabilization; -Biofouling and microbial metal corrosion;	- Rougeaux et al., 2001; Goh et al., 2009; Moppert et al., 2009; Benninghoff et al., 2016; Flemming et al., 2016; - Paterson et al., 2008; Gerbersdorf et al., 2009; Grabowski et al., 2011; Yang et al., 2016; -de Nys et al., 2009; Camacho-Chab et al., 2016;
Optical transparency	- Enhanced forward-scattering of photons;	-Decho et al., 2003;
Protection	-Hydrothermal vents; -Protection from grazing; -Antifreeze protection	-Rougeaux et al., 2001; Guezennec, 2002; -Plante, 2000; DePas et al., 2014; -Marx et al., 2009; Underwood et al., 2010; Liu et al., 2013; Ewert and Deming, 2014; Boetius et al., 2015;

*CNN, cloud condensation nuclei; *e*-enzymes, extracellular-enzymes; DOM/POM, dissolved/particulate organic matter; MOS, marine oil snow; TEP, transparent exopolymer particles; UV, ultraviolet; ^∗^references are examples and not all-inclusive.*

Finally, it is important to note that in ocean systems, the microbial communities of aggregates suspended in the water-column, and the sea-surface slick communities of oceans are also biofilms, since these communities contain EPS, and exhibit differing levels of organization. EPS are also secreted as a ‘metabolic by product.’ These are most apparent during the later stages of phytoplankton blooms, and will be discussed further below. Taken together, microbial extracellular secretions are now thought to comprise a large portion of the bioavailable carbon pool in oceans, especially in dissolved forms. The total amount of microbially produced EPS, although difficult to measure accurately and precisely, is likely to be very substantial.

## Dissolved and Particulate Organic Carbon in the Ocean

Organic matter in seawater constitutes a complex mixture of compounds in a dissolved and particulate form – respectively, dissolved organic matter (DOM) and particulate organic matter (POM). Both forms serve a source of carbon and nutrients to heterotrophic microorganisms, including to mixotrophic eukaryotic phytoplankton and filter feeders. DOM is the dominant form of carbon in the oceans that can originate from any number of sources, much of which is produced *in situ* by marine microorganisms (largely eukaryotic phytoplankton and bacteria) and is derived from terrestrial inputs via transportation from river effluents and surface runoff. DOM comprises up to 700 Gt of carbon in the ocean, which is a staggering amount of dissolved organic carbon (DOC); so much so that 1% annual change of it in the ocean can produce as much CO_2_ as that from fossil fuel combustion per annum (Hedges, 2002). Up to 70% of DOM in the oceans averages a molecular weight of <1 kDa and is defined as low-molecular-weight DOM (Benner, 2002), the bulk of which is refractory (Bauer et al., 2002) and difficult to chemically characterize down to the molecular level. The high-molecular-weight fraction of DOM (>1 kDa) in the oceans contributes about 30% of DOC. It is more labile and thus more readily degraded (Amon and Benner, 1994; Guo et al., 1994).

Depending on its physical state in seawater (gel, colloidal, or particulate form), DOC/POC can serve as a surface to which microorganisms attach. Marine snow, which comprises aggregates of >500 μm, is formed in the upper water-column when dead and dying phytoplankton cells come together with other planktonic microorganisms within a matrix of biopolymers (Alldredge et al., 1993; Tiselius and Kuylenstierna, 1996). Marine snow is one form of POC that is a key component of the biological pump in the ocean that participates in the redistribution of carbon in marine systems and principally in the flux of fixed carbon to the sea floor (Shanks and Trent, 1980; Shanks and Reeder, 1993; Long and Azam, 2001b). The processing of organic matter, such as marine snow, by bacteria in the ocean significantly affects its vertical flux from the upper water column to the ocean floor, and in turn impacting the global cycling of carbon and the planet’s climate (Simon et al., 2002). The transport of organic carbon via sinking of POC from the sea surface to the seafloor is another major component of the “biological pump,” which globally contributes in the exports of ca. 10 Gt C per year from the euphotic zone and accounts for 20% of ocean primary production (Treguer et al., 2003). However, at depths approaching 2000 m, this flux or organic carbon decreases to about 1% as the other 19% is mineralized and cycled by the “microbial loop.”

In oceanography, organic matter in seawater is operationally defined as “dissolved” (i.e., DOM) if it passes through a 0.7 μm pore size filter; that which is retained on the filter is defined as POM. The diversity of dissolved organic carbon in seawater ranges from ‘truly’ dissolved molecules, such as glucose, to colloidal and transparent gel-like matter, and can also include microorganisms (e.g., micro-algae, bacteria, archaea, viruses) if they too pass through a 0.7 μm pore size filter. The introduction of sensitive analytical techniques for analyzing seawater, such as high-performance liquid chromatography (HPLC) (Mopper et al., 1992) have increased our understanding of the major classes of DOM in the ocean. Methods to recover and characterize DOM and POM are described by Wurl (2009).

### Water Column

#### DOC and POC

The world’s oceans contain a total DOC content that is comparable in mass to the carbon in atmospheric CO_2_ (Hansell and Carlson, 1998). The oceanic DOC pool comprises a wide spectrum of compounds, much of which is chemically uncharacterized – it could be regarded as a ‘black hole’ in terms of our relatively poor understanding of its chemical composition and from what biogenic sources this massive pool of organic carbon molecules originate. At least among the chemical constituents of oceanic DOC that have been characterized, three major compound classes have been identified: carbohydrates (mono- and polysaccharides or EPS), proteins, and lipids. Much of the DOC in the ocean water column exists as EPS biopolymers (ca. 10–25% of total oceanic DOM) that undergo reversible transition between colloidal and dissolved phases (Verdugo, 1994; Chin et al., 1998). Based on its predominance throughout the world ocean, it has important implications in microbial interactions and biogeochemical cycles.

#### Extracellular Polymeric Substance

The synthesis and extracellular release of EPS by eukaryotic phytoplankton and bacteria forms a major component to the total DOC pool in the ocean (Verdugo, 1994; Aluwihare et al., 1997). EPS can serve a variety of functions, such as in the binding and fate of trace metal-nutrient species, the solubilisation of hydrophobic organic chemicals, and in biofilm formation (Decho, 1990; Santschi et al., 1998). Compared to EPS produced by marine eukaryotic phytoplankton (Bhaskar and Bhosle, 2005) and non-marine bacteria (Ford et al., 1991), EPS produced by marine bacteria generally contains higher levels of uronic acids, notably D-glucuronic and D-galacturonic acid (Kennedy and Sutherland, 1987). This renders these macromolecules highly polyanionic (negatively charged), which may be attributable to any number of anionic groups (e.g., COO^-^, C–O^-^, SO_4_^-^) and consequently quite reactive in their potential to interact with other chemical species (Kennedy and Sutherland, 1987). Nonetheless, the EPS released by some eukaryotic phytoplankton species can also be rich in uronic acids, such as that produced by the coccolithophore *Emiliania huxleyi*, which contains up to 20% galacturonic acids of total sugar content (De Jong et al., 1979).

The polyanionic nature of EPS serves important ecological functions in marine systems. These include microbial adhesion and biofilm formation (Thavasi and Banat, 2014), the emulsification of hydrocarbon oils and influencing their biodegradation (Gutierrez et al., 2013), or mediating the fate and mobility of heavy metals and trace metal nutrients (Bhaskar and Bhosle, 2005; Gutierrez et al., 2008, 2012). This wide spectrum of functional activity is reflected not merely in the complex chemistry of these molecules, but also in the diversity of bacterial genera producing them (Thavasi et al., 2011). Overall, the composition of marine EPS varies due to the producing species and physiological stage (Myklestad, 1977; Grossart et al., 2007).

A number of reports have described marine bacterial EPS binding heavy and toxic metal ions such as Cd, Cr, Pb, Ni, Cu, Al, and Ur (Zosim et al., 1983; Beech and Cheung, 1995; Schlekat et al., 1998; Iyer et al., 2005; Bhaskar and Bhosle, 2006; Gutierrez et al., 2008). Whilst the rationale to many of these studies was commercial, a few have addressed the ecological implications of marine EPS in biogeochemical cycles. In two studies by Loaec et al. (1997, 1998), the authors reported on the heavy metal-binding capacity of EPS produced by hydrothermal vent bacteria, and showed that this might represent a survival strategy for the bacteria by reducing their exposure to toxic metals released from the hydrothermal vents. Major elemental constituents of seawater, such as Na, Mg, Ca, K, Sr and Si, have been shown to be adsorbed by marine bacterial EPS (Gutierrez et al., 2008). What ecological implications this may have in marine systems, or indeed to the producing organisms, remains to be more-fully understood.

A key role of polyanionic EPS, particularly in the euphotic zone, is in its potential role in controlling soluble iron (Fe^3+^) bioavailability. Studies in recent years have shown single anionic residues, such as glucuronic and galacturonic acids (Hassler and Schoemann, 2009; Hassler et al., 2011b), and purified marine bacterial EPS containing high levels of uronic acids (Gutierrez et al., 2008; Hassler et al., 2011a), can effectively bind Fe^3+^ and promote the uptake of this trace metal by eukaryotic phytoplankton (Hassler et al., 2011b; Gutierrez et al., 2012). The implications of this are significant because of the abundance of EPS in the ocean (Verdugo et al., 2004) and because Fe^3+^ is an essential trace metal that limits primary production in up to 40% of the open ocean (Martin et al., 1994; Boyd et al., 2007).

A large fraction of the EPS produced by bacteria in the ocean is of glycoprotein composition (Long and Azam, 1996; Verdugo et al., 2004). The amino acid and peptide components found associated with these glycoprotein biopolymers have been shown to confer amphiphilic characteristics to these macromolecules (Verdugo et al., 2004; Gutierrez et al., 2009), and which could explain, at least in part, their ability to interact with hydrophobic species, such as oil hydrocarbons.

#### Transparent Exopolymer Particle

A special class of EPS that are described as mucopolysaccharides is transparent exopolymer particles (TEPs). It is operational defined based on being retained by a filter with a pore size of >0.4 μm (Alldredge et al., 1993), and based on this, TEP are defined as gel particles. TEP exists in the water column suspended in colloidal form, likely formed via the aggregation of smaller EPS molecules (Engel et al., 2004). Aggregation may be mediated by the bridging of divalent cation (Ca^2+^, Mg^2+^) and half-ester sulfate (OSO_3_^-^) moieties of acidic monomers that constitute individual EPS molecules. TEP is transparent, but because these gel particles are rich in acidic sugars they can be observed under the light microscope after staining with the cationic copper phthalocyanine dye Alcian Blue at pH 2.5 (Alldredge et al., 1993).

The abundances of TEP in the ocean water column are on average in the order of 10^6^ per L of seawater, and can reach as high as 10^8^ per L (Passow, 2002; Bhaskar and Bhosle, 2005), particularly during periods of phytoplankton blooms. The contribution of TEP to the pool of POC in the upper water column in the Atlantic and Adriatic during certain periods of the year has been shown to be quite significant (Engel and Passow, 2001). A fraction of the TEP pool in the ocean is proteinaceous. It is referred to as Coomassie stainable particles (CSPs) because these gel particles can be stained with the amino acid-specific dye Coomassie Brilliant Blue and observed under the light microscope (Long and Azam, 1996). The abundances of CSP in coastal waters range between 10^6^ and 10^8^ per L of seawater (Long and Azam, 1996).

Transparent exopolymer particle contribute significantly to what is described as the marine gel phase. Verdugo et al. (2004) suggested this phase to span a large size spectrum, from colloids to particles of several 100s of micrometers. Its formation has been described to originate from the spontaneous aggregation of DOM molecules into POM within minutes in seawater (Chin et al., 1998) – a process that may involve crosslinks facilitated by cation bridging between DOM molecules.

#### Microbial Associates

Particulate organic matter can be described as a “hot spot” for microbial (esp. bacterial) activities in the water column, containing a rich microbial community with abundances reaching up to two orders of magnitude higher than in the surrounding seawater environment (Alldredge et al., 1986; Herndl, 1988). The establishment of a bacterial community within and surrounding (biofilm) POM leads to various levels of microbial interaction that include mutualism and antagonism (Long and Azam, 2001a), as well as cooperative behavior such as QS (Gram et al., 2002). A study assessing the phylogenetic diversity of POM-associated versus free-living bacteria from a site ca. 5 km offshore the Santa Barbara coast revealed distinct differences between these communities, with primarily members of the *Cytophaga, Planctomyces*, and *Gammaproteobacteria* dominating aggregate particles, whereas *Alphaproteobacteria* dominated the free-living fraction (DeLong et al., 1993). Bacteria associated with POM have been shown to exhibit high activities for a range of extracellular enzymes (Hoppe et al., 2002; Simon et al., 2002), likely contributing to the hydrolysis of the POM aggregates. Whilst rich in microbial diversity and abundance, POM accounts for only <10% of total bacterial abundance and production in the marine water column, with the majority of bacterial cells occurring in a free-living state.

Studies using oxygen microelectrodes to measure dissolved oxygen in POM aggregates have shown that even the tiniest of marine snow particles can contain anoxic environments (Alldredge and Cohen, 1987; Alldredge and Silver, 1988; Ploug et al., 1997; Ploug, 2008). The high extracellular enzyme activities by bacteria associated with POM will deplete oxygen concentrations that create anoxic micro-niches within the aggregates, potentially supporting the growth of obligate anaerobic or microaerophilic microorganisms (Bianchi et al., 1992). It may therefore, be expected that diverse aerobic and anaerobic microorganisms associated with marine snow aggregates would colonize different niches of the aggregates. The formation of an oxygen gradient, which is increasingly more anoxic toward the interior of the aggregates, would pose a strong influence on the stratification of the microbial community. Essentially, the interior of aggregates will be enriched with obligate and/or facultative anaerobes.

### Air–Water Interface

#### Surface Water Droplet Formation, Sea Spray, and Cloud Formation

Biological processes on the sea surface of the ocean can have a direct effect on atmospheric processes, such as modulating CO_2_ exchange and release of cloud condensation nuclei (CCN), that in turn influence the Earth’s climate. CCN are atmospheric particles that serve as nuclei for the formation of cloud droplets by taking up water vapor because they are sufficiently soluble. In the past decade there has been an increasing body of evidence supporting the hypothesis that atmospheric marine aerosols contain the same organic species that are found in oceanic DOM (Leck and Bigg, 2005; Bigg, 2007; Facchini et al., 2008). Seawater DOM, much of which comprises phytoplankton exudates and bacterial EPS, can be ejected into the atmosphere when bubbles at the sea surface burst (Bigg, 2007; Leck and Bigg, 2008). Bubbles can form by a number of physical forces at the sea surface, ranging from raindrops to breaking waves, which then burst and produce submicron droplets that disperse as aerosol into the atmosphere and carrying with it marine organic species such as microbial cells and DOM. A study by Kuznetsova et al. (2005) showed that TEP and CSP can accumulate in the sea surface microlayer and subsequently, through bubble bursting, become transported to the atmosphere as marine aerosol. The authors showed that the aerosols contained a large number of semitransparent gel-like particles, in addition to microorganisms, organic and inorganic matter. The semitransparent gel-like particles (primarily TEP and CSP) in the aerosols all contained amino acids, and based on D/L ratios of these acids it was suggested that they originated from phytoplankton exudates.

Several studies have shown that the organic species entrained within marine aerosols collected from various remote ocean sites are of a size range between 70 and 200 nm in diameter. The dominant size range between 50 and 100 nm (Tyree et al., 2007; Fuentes et al., 2010; Hultin et al., 2010), is reminiscent of EPS gels found on the sea surface (Bigg, 2007; Bigg and Leck, 2008; Leck and Bigg, 2008). In a review by Hawkins and Russell (2010) covering over 10 years of measurements of ocean-derived aerosol, the authors concluded that the organic species within marine aerosol is composed of EPS, proteins and amino acids, as well as microorganisms and their components. Facchini et al. (2008) suggested that the solubility continuum of phytoplankton exudates found in seawater is also reflected in marine aerosol, and there is a growing body of evidence supporting the hypothesis that phytoplankton exudates contribute to the formation of CNN (O’Dowd et al., 2004; Russell et al., 2010). Upon its entry into the atmosphere through bubble bursting, the entrained organic gel aggregates within the aerosol particles either directly contribute to the CCN pool in the marine boundary layer (MBL) or after they are degraded by ultraviolet light or acidification in the atmosphere.

Recent research by the DROPPS consortium, funded through the Gulf of Mexico Research Initiative (GOMRI) program, is carrying out experiments attempting to recreate the sea surface microlayer to investigate the potential for petrocarbon (crude oil) to enter the atmosphere. Initial results of this work reveal that crude oil droplets, formed by treatment with dispersants, can burst through physical forces and form aerosolized droplets containing crude oil. This oil-containing aerosol could be carried long distances by wind in the atmosphere and potentially pose health threats to humans and wildlife when inhaled or upon coming in contact with skin.

#### Marine Oil Snow

Marine oil snow (MOS) is essentially marine snow, with the exception that it distinctively contains oil hydrocarbons. Current knowledge recognizes its formation to be confined to the sea surface where oil slicks form in the event of an oil spill, but further work is needed to determine if MOS can also form in the subsurface. MOS can be described as a mucilaginous organic matter with a “fluffy” or gelatinous off-white appearance that contains oil droplets embedded within its amorphous matrix. Previous reports described evidence of MOS formation during the Ixtoc-I (Boehm and Fiest, 1980; Jernelöv and Lindén, 1981; Patton et al., 1981) and *Tsesis* (Johansson et al., 1980) oil spills (Teal and Howarth, 1984). However, MOS only recently received considerable attention when copious quantities of it, of macroscopic cm-size dimensions, were observed within 2 weeks of the Deepwater Horizon (DWH) blowout in the Gulf of Mexico – a spill recorded as the worst oil spill disaster in US history. MOS was encountered frequently around the vicinity of surface oil slicks at DWH (Niu et al., 2011; Passow et al., 2012). It eventually sank to the seafloor in the Gulf of Mexico – a process described termed MOSSFA (Marine Oil Snow Sedimentation and Flocculent Accumulation), which contributed a significant role in the export of crude oil (ca. 14% of the oil released at DWH) to the sediment (Valentine et al., 2014).

**FIGURE 2:**
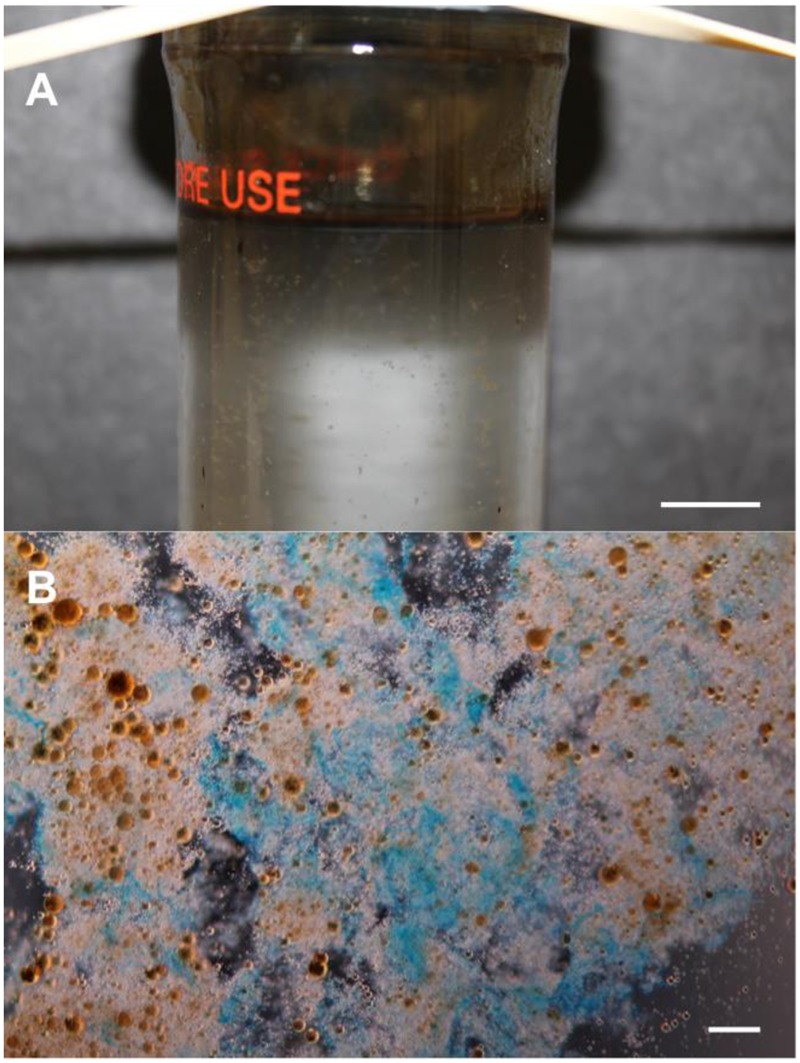
**Formation of marine oil snow (MOS). (A)** A roller-bottle incubation showing the formation of MOS in synthetic seawater amended with crude oil and inoculated with the EPS-producing (and oil-degrading) bacterium *Alteromonas* sp. strain TK-46(2) that was isolated from sea-surface oil slicks during the Deepwater Horizon oil spill. **(B)** Light micrograph of MOS aggregates, after staining with the cationic copper phthalocyanine dye Alcian Blue showing that the aggregates are partially composed of polysaccharide. The orange-brown spheres in **(B)** are emulsified oil droplets embedded within and adsorbed to the amorphous matrix of the aggregates. Scale bars = 10 mm **(A)**, and = 10 μm **(B)**.

Conjecture still surrounds MOS genesis at DWH and during the Ixtoc-I and *Tsesis* oil spills, but its formation and sedimentation appears to have been directly associated with the influx of crude oil. In roller-bottle experiments performed under conditions attempting to simulate sea surface oil slicks at the DWH spill, the presence of crude oil was shown to be an important factor in triggering MOS formation, and that MOS acted as hotspots for microorganism and oil-degrading enzyme activities (Ziervogel et al., 2012; Gutierrez et al., 2013). Bacterial and eukaryotic phytoplankton cells and/or their produced polymers (e.g., EPS) have been reported to induce MOS formation (Passow et al., 2012; Gutierrez et al., 2013; Passow, 2016), whilst there are reports describing conflicting results on the role of dispersants in this respect (Baelum et al., 2012; Fu et al., 2014; Kleindienst et al., 2015; Passow, 2016; Suja et al., 2017). **Figure 2A** shows MOS formation in a roller-bottle incubation containing synthetic seawater amended with crude oil and *Alteromonas* sp. strain TK-46(2) – an oil-degrading and EPS-producing bacterial strain that was found enriched in surface oil slicks in the Gulf of Mexico during the DWH spill (Gutierrez et al., 2013). Like TEP, MOS particles can be rich in acidic sugars of polysaccharides, such that may be produced by EPS-producing bacteria like strain TK-46(2) (**Figure 2B**).

Despite the interest in MOS formation as a product of spilled oil into the Gulf of Mexico, the microorganisms associated with MOS particles have received less attention. During incubations with uncontaminated deep-water samples collected during the active phase of the DWH oil spill and amended with the dispersant Corexit, Baelum et al. (2012) reported the formation of MOS, which was dominated by members of the genus *Colwellia*. In a more in-depth study of the bacterial community associated with MOS, Arnosti et al. (2015) showed that MOS particles contained a bacterial community that was distinctly different from that found freely living (i.e., not associated to MOS) in the surrounding seawater environment. The MOS-associated community was dominated by oil-degrading and EPS-producing members of the *Gammaproteobacteria*, principally *Cycloclasticus, Congregibacter, Haliela, Halomonas* and *Marinobacter*, and included diverse members of the *Alphaproteobacteria* (principally the *Roseobacter* clade) and some members within the *Bacteroidetes* and *Planctomycetes*. Using CARD-FISH (catalyzed reporter deposition – fluorescence *in situ* hybridization), MOS particles formed in incubations with Macondo crude oil and the dispersant Corexit were dominated by members of the class *Gammaproteobacteria*, including the order *Alteromonadales*, which comprises oil-degrading and EPS-producing taxa (Kleindienst et al., 2015). Using Illumina MiSeq sequencing, Suja et al. (2017) showed MOS particles formed in subarctic waters to be enrichment with oil-degrading (*Alcanivorax, Cycloclasticus, Thalassolituus, Marinobacter*) and EPS-producing (*Halomonas, Pseudoalteromonas, Alteromonas*) bacteria, and included major representation by *Psychrobacter* and *Cobetia* with putative oil-degrading/EPS-producing qualities. Collectively, these studies indicate that MOS are hotspots where oil-degrading and EPS-producing bacteria are enriched, and the latter may provide a clue on the role of these organisms in MOS formation through their synthesis and release of ‘sticky’ EPS.

Whilst significant knowledge gaps exist in our understanding on MOS formation and its subsequent sedimentation to the sea floor, the influx of crude oil and its interaction with planktonic microorganisms, as well as with dissolved and colloidal organic polymers, such as TEP, and with nutrient and suspended mineral discharges from river effluents, appear to be important factors that warrant further investigation (see Daly et al., 2016 for a review).

### Turnover and Stability

Dissolved organic carbon in the ocean can be classified into three broadly defined pools of carbon based on their turnover times: labile, semilabile, and refractory. Collectively, the concentrations of these three DOC pools typically range from 60 to 90 μmol/L in the upper ocean water column, decreasing with depth to 40 μmol/L in the deep sea (Hansell, 2002). Fluctuations in the concentration of DOC in the water column occurs over certain periods of the year, due largely to periods of elevated photosynthetic production since this is the main source that fuels each of these three classes of DOC. The combined effect of biological and physical processes that alter the concentrations of these three pools of DOC is represented in the size-reactivity-continuum model (Amon and Benner, 1996; Benner and Amon, 2015), for which microbial processing of these molecules is the major mechanism that leads to rendering them progressively more recalcitrant (Jiao et al., 2010).

#### Labile DOC

Labile DOC in seawater comprises substrates with short residence times (minutes to days) since they are consumed almost as quickly as they are produced or released into the water column. An example of a common labile substrate in seawater is glucose, which is on average found at concentrations from 0.001 to 1.0 μmol/L, depending on the ocean region (Rich et al., 1996, 1997; Benner, 2002; Skoog et al., 2002). Other mono-sugar substrates (monosaccharides) also exist in the water column at concentrations typically ranging from 0.002 to 0.8 μmol C/L (Benner, 2002). On average across the oceans, glucose is the most abundant simple sugar, with concentrations as high as 187 nM measured in unfiltered water of the Gulf of Mexico, and 490 nM in high-molecular-weight DOM of the equatorial Pacific (Skoog and Benner, 1997). Glucose has been shown to contribute significantly in supporting a major fraction of bacterial growth in many ocean systems (Rich et al., 1996, 1997; Grossart and Simon, 2002), though other studies have shown glucose to play a less significant role in this respect (Keil and Kirchman, 1999; Skoog et al., 1999, 2002; Kirchman et al., 2001). Turnover rates for glucose can depend on the ocean environment, including the availability of certain nutrients, varying from rapid (hours to days) to relatively slow (100s of days). For example, surface waters limited by inorganic phosphorous can limit bacterial consumption of labile DOC, such as glucose, and result in a longer-than-average residence time of the endogenous pool of the labile DOC (Thingstad et al., 1997).

With respect to the chemical composition of POC in seawater, the abundance of glucose may be related to the major roles that this monosaccharide plays in phytoplankton biology – polymers of glucose (glucans) are major storage compounds in phytoplankton. Galactose is the second most abundant sugar in seawater, and polymers of it (galactans) are major structural components of phytoplankton cell walls (Romankevich, 1984).

Another simple carbohydrate that also contributes to the total pool of labile DOC in the ocean is mannitol. It is one of the most abundant sugar alcohol compounds in nature (Stoop et al., 1996); it is found in bacteria, fungi, algae and higher plants, where it often acts as a compatible solute, among conferring other functions. In ocean systems, mannitol is a major product of photosynthetic organisms, like algae, whereupon this polyol is released following cell lysis to join the pool of labile DOC in the ocean.

Carbohydrate concentrations in seawater can be as high as 10 μmol/L and contribute a significant fraction to the pool of labile substrates. Dissolved polysaccharides, such as EPS produced by bacteria and algae, form a major fraction of the total carbohydrates in the water column (Benner, 2002). Since concentrations of monosaccharides are typically 10-fold lower than dissolved polysaccharides, this suggests they are likely cycled more rapidly. Polysaccharides nonetheless contribute to fueling a major fraction of bacterial activity in some marine environments. On average, however, concentrations of labile DOC are very low (<1 μmol/L), constituting less than 1% of total organic carbon in the upper water column of the ocean. These substrates could potentially sustain oligotrophic microbial populations in regions of poor nutrient availability, such as in the open ocean. Nonetheless, the labile DOC pool is continuously replenished on a yearly basis by trophic (phytoplankton and bacterial excretion) and non-trophic (viral lysis, grazing) processes (Nagata, 2000).

#### Semilabile DOC

Approximately half of the total pool of DOC in the upper ocean water column is classed as semilabile, and comprises substrates that are consumed over weeks to months. Concentrations of semilabile DOC typically range from 20 to 30 μmol/L in the ocean water column, and because it is consumed over this median time scale, it assumes that this pool of DOC is important in supporting bacterial growth over seasonal to annual time scales (Carlson et al., 1994; Repeta and Aluwihare, 2006). In some ocean systems, such as the Sargasso Sea, the total semilabile DOC can account for as much as 89% of the total DOC (Carlson et al., 1994). In this study, up to 50% of this semilabile DOC was found to be more resistant to microbial degradation over weeks to months.

Interestingly, DOC produced in high-nutrient environments has been observed to be less susceptible to microbial degradation than that produced in low-nutrient environments. This may relate to the chemical qualities of the DOC produced in these contrasting environments – DOC produced in high-nutrient waters may be more nutrient rich than that produced in low-nutrient waters (Church, 2008). Semilabile DOC can accumulate as a result of inorganic nutrient limitation of bacterial growth (Thingstad et al., 1997), primarily from a limitation in ${\mathrm{PO}}_{4}^{3-}$ (Cotner et al., 1997; Rivkin and Anderson, 1997; Thingstad et al., 1998; Zohary and Robarts, 1998; Caron et al., 2000). Other studies, however, have not found evidence to support the hypothesis that inorganic nutrient limitation of bacterial growth leads to accumulation of semilabile DOC. Rather, the chemical nature of this DOC class, specifically acting as a poor substrate for bacterial degradation, likely contributes to its accumulation in the water column. This is especially the case in the upper water column where DOC concentrations are higher than in the mesopelagic. This could influence the microbial communities in these contrasting regions of the water column where microbes in the upper water column have been found to degrade semilabile DOC less rapidly than those communities found in the mesopelagic (Carlson et al., 2004).

#### Refractory DOC

Much of the DOC in the ocean consists of low-molecular-weight solutes of <1000 Da, the majority of which comprises the refractory pool of DOC that is resistant to microbial degradation over time scales of 1000s of years – anywhere between 4000 and 6000 years (Williams and Druffel, 1987) – approaching or exceeding that of ocean circulation (Benner et al., 1992). Whilst concentrations of labile and semilabile DOC vary with depth, that of refractory DOC averages ca. 40 μmol/L throughout the water column with little to no variation with depth, and likely contributes insignificantly as a source of carbon and energy to bacterioplankton. However, a fraction of refractory DOC at the sea surface is destroyed by ultraviolet irradiation, which results in the release of labile DOC for heterotrophs to consume (Moran and Zepp, 2000). The refractory pool contributes to the sequestration of enormous quantities of carbon and acts as a carbon sink in the water column (Hedges, 2002). A recent study combining organic matter size, ^14^C age and elemental composition of DOC estimated that small refractory molecules in the ocean are produced by microorganisms and at a rate of 0.24 PgC per year, which is on par in magnitude to the burial of organic carbon in sediments (Walker et al., 2016).

Major sources of this refractory DOC pool include complex cell fragments and other high-molecular-weight biopolymers produced by cells that are partially or almost totally recalcitrant to biodegradation. During viral lysis of eukaryotic phytoplankton cells, cellular components that are highly refractory to degradation are released into the water column. Similarly, phage-mediated lysis of bacterial cells leads to the release of outer-membrane proteins of the cell envelope, which also are very resistant to degradation. Conversely, highly labile cell components, such as nucleic acids and amino acids, are recycled in the photic zone.

A proportion of the DOC released through cell lysis, including that produced extracellularly by living cells, is converted by chemical mechanisms into humic substances. This complex material is quite resistant to biodegradative processes and contributes to the total pool of refractory organic matter in the water column. Either by agglomeration of these humic substances, or their attachment to other sinking particles, a proportion of this refractory material eventually sinks to the ocean floor and becomes buried. Not all refractory DOC in the marine water column, however, sinks to the seafloor. Rather, most of it remains suspended and circulating in the ocean for years to millennia. In fact, of the fixed organic carbon formed by primary production, only a very small fraction reaches the seafloor; much of it (>99%) is remineralized in the water column through microbial action.

Extracellular polymeric substance produced by marine bacteria, even that which is freshly produced, can be somewhat refractory to microbial degradation or chemical analysis (Ogawa et al., 2001). This is believed to be related to the presence of uronic acids (Anton et al., 1988; Bejar et al., 1996) or glycosidic linkages of hexosamines (Biermann, 1988). Such constituents can confer on EPS molecules a high resistance to degradation under acid hydrolysis conditions that are used to chemically analyze them. Some studies analyzing the chemical nature of EPS isolated from cultured marine bacterial strains have shown a major proportion of these macromolecules (up to 80%) can be unaccounted for by chemical analysis (Gutierrez et al., 2007a,b, 2008).

## Physical/Chemical Properties of EPS

Extracellular polymeric substance comprise an expanding plethora of biochemical molecules that interact in many ways and that are, as yet, poorly understood. EPS consist of an array of molecules, ranging from quite large (e.g., >100 kDa) to much smaller (e.g., <10 kDa) polymers. Some of the molecules contribute to the structural stability, gel properties, and pliancy of the greater matrix (Flemming, 2016). We must determine how different types of molecules in this matrix interact with each other in order to provide the observed functional roles of the EPS matrix. An entire rethinking of the extracellular milieu of microorganisms will likely be required. An insightful examination is provided by Neu and Lawrence (2016).

### Composition and Physical Properties

From the standpoint of microbial cells, the EPS, especially when in a gel state, form a three-dimensional matrix or scaffold within which cells can orient themselves relative to one another. The presence of certain polymers can afford the matrix physical stability. Polymers such as amyloids and/or eDNA (discussed below) can serve as an architectural framework for the EPS. Each type of EPS component can offer different physical properties (Chew et al., 2014). The physical ultrastructure of the EPS matrix has been very difficult to image in a fully hydrated state (Decho, 1999), owing to its delicate nature. Excellent pioneering efforts have been conducted by Dohnalkova et al. (2011) using a unique cryo-TEM approach. Recent developments in cryo-TEM and -SEM may provide further insights into this complex matrix.

Bacterial and microalgal EPS exist in nature in a range of different physical states, most of which are operationally defined. Capsules consist of polymers that closely surround individual or multiple cells and often serve a protective role (Whitfield, 2006). Further away from cells, EPS can exist as tight, dense-gels, to a continuum of physical states from looser-slime to truly dissolved forms. Dissolved forms, in the absence of cells, may condense to microgels in the open ocean (Chin et al., 1998; Verdugo et al., 2004; Verdugo and Santschi, 2010); a process that is of significant importance and has been well-summarized in a review (Verdugo, 2012). While differences in these physical states are relatively arbitrary, they likely serve very different functions to the microbial cells secreting them. The physical state of EPS results from a combination of the polymer concentrations, types and abundances of ions, composition, and steric availability of functional groups on polymers. Recently, this has been reviewed in greater detail (Neu and Lawrence, 2016).

Some microbial EPS can exhibit significant viscoelastic properties, being able to stretch and retract in response to an applied force such as intermittent water flow (Fabbri and Stoodley, 2016). This property offers the microbial cells contained within a biofilm with a certain degree of physical flexibility and mechanical resiliency (Peterson et al., 2015). This flexibility is important to the ability of cells to persist on a surface or as an aggregate in the water-column.

Compositional information is a useful starting point for investigating EPS. Strictly speaking, the term EPS is an operational designation that refers to the milieu of ‘larger’ molecules contained in the extracellular matrix in proximity to cells (Wotton, 2004). Many of the molecules contained in natural EPS cannot be conveniently characterized as either protein, lipid or carbohydrate. For this reason, it has been called the ‘*dark matter*’ of biofilms (Flemming et al., 2007). It is now known that the EPS matrix can be quite heterogeneous, especially over small spatial scales (see Microdomains within EPS Matrices). It is for this reason that analyses of ‘bulk’ samples will not capture the smaller-scale variability that are critical to understanding the physical and chemical properties of the *in situ* matrix. When EPS from natural biofilms, for example, are extracted and then reconstituted, the physical (e.g., gel, viscocity, rheology, etc.) properties of the reconstituted EPS often do not readily resemble those of the original biofilm. (In much the same way, a cell cannot be taken apart via extractions, and then put back together as a functioning cell.) This suggests that the molecules within the matrix may have important molecular organization either by purposeful design or by result of the environment. *It is important from a functional standpoint, to determine which molecules and molecular interactions contribute to physical properties such as gel formation, rheology, and diffusion-slowing, and chemical properties, such as sorption*.

Finally, while matrix is actively secreted by cells, the properties of EPS may be changed post-secretion, modified by geochemical, enzymatic, and photochemical processes, and additionally contain trapped or sorbed molecules. These modifications may have dramatic effects on their physical properties. Thus, EPS under natural conditions exist in a ‘continuum’ of compositional and partial degradation states. Thus, it is imperative that future non-destructive approaches (e.g., Raman spectroscopy) are developed for characterizations of EPS *in situ*.

Laboratory studies of bacteria secreting EPS show that cells typically produce sugar monomers that are exported, then assembled to the existing polymer outside of the outer membrane (Whitfield, 2006; Sutherland, 2016). Polymers may consist of single sugar monomers, called homopolymers, or consist of several monomers linked together to form a repeating unit, called heteropolymers. Since several types of repeating units may be generated, this provides the cell with the capability to alter the physical chemical properties by mixing different amounts of repeating units. By changing the building blocks (i.e., repeating units), the polymer composition can be varied. This allows the polymer to have a variable composition and provides the cell the capability to modify its extracellular polymers in response to changing conditions.

At present, there are no specific biosignatures that can be assigned reliably for detection of EPS, simply because similar glycol-based compounds are produced throughout biological systems for many purposes. The secretion of carbohydrates and other glycosylated polymers is not unique to bacteria or even microorganisms; rather, it is a universal biological strategy employed by microalgae, fungi, invertebrate and vertebrate animals, and plants (Underwood and Paterson, 2003).

Extracellular polymeric substance have often been considered synonymous with ‘exopolysaccharides’ and acronym EPS. This was partially based on the carbohydrate-focus of investigators at the time, and additionally due to the artifact of carbon-rich culture conditions that were used to grow bacteria and obtain abundant quantities of EPS. Much has been learned regarding polysaccharide chemistry from these seminal studies. While polysaccharides are a major component of many natural EPS, they are only a component. The EPS matrix is now known to contain several different major groups of molecules, whose roles and involvement are still under study, and will be discussed briefly below. Hence, much discussion has evolved about what exactly constitutes the EPS matrix. Three points emerge as one studies the EPS of natural systems: (1) Many different types of molecules interact to provide the physical structure, impart chemical properties, and even actively manipulate EPS properties for the cell; (2) EPS-compositional studies should not be limited to culture-based systems; and (3) EPS under natural conditions will likely exist in a continuum of partial-degradation states.

#### Polysaccharides

The polysaccharide components of EPS are perhaps the best-studied to date. Common carbohydrate components that are often found in EPS include monomers such as D-glucose, D-galactose, D-mannose, L-fucose, L-rhamnose, D-glucuronic acid, D-galacturonic acid, L-guluronic acid, D-mannuronic acid, *N*-acetyl-D-glucosamine, and *N*-acetyl-D-galactosamine (Sutherland, 2001, 2016). Polysaccharides such as cellulose, alginic acid, dextran, xanthan, and *Vibrio* exopolysaccharide (VPS) are examples of polysaccharides produced by bacteria (for reviews see Decho, 1990; Wotton, 2004; Serra et al., 2013; Hobley et al., 2015).

The exopolysaccharide portion can exert significant net effects on physical and sorptive properties of EPS (Salek and Gutierrez, 2016). The presence of polar negative-charged groups such as carboxyls, phosphates and sulfate esters can provide negative charges (Thornton et al., 2007; Gutierrez et al., 2009). In certain microbial mat systems, highly sulfated exopolysaccharides have been isolated, and contained up to near 30% (wt/wt) in sulfate (Moppert et al., 2009).

In the presence of (positive) divalent cations (e.g., Ca^2+^, Mg^2+^) they can form cation bridges with adjacent polymers having also negative functional groups. EPS having abundant uronic acids often complex in this manner. Direct linkages between adjacent EPS can also occur. For example, linkages between a cationic polysaccharide and (anionic) extracellular-DNA has been shown to contribute to the physical stability of bacterial biofilms (Jennings et al., 2015). The abilities of EPS to link with each other is dependent, in part, on pH, the presence of appropriate functional groups, and the steric availability of functional groups (Decho, 1990; Ulrich, 2009).

#### Microdomains within EPS Matrices

The natural EPS matrix is now known to be heterogeneous over small spatial scales (e.g., μm, even nm). In order to describe small, localized areas that exhibited different properties than the broader matrix, polysaccharide chemists long ago developed the term ‘*microdomain.*’ The microdomain concept was extended to EPS (Decho, 2000a) to begin understanding why intact EPS exhibited very different properties than those of the extracted bulk EPS. Microdomains can result from localized concentrations of certain monomeric components of polymers, and/or differential binding of adjacent polymers to each other. Evidence for microdomains within *in situ* expolymer matrices has been evidenced by the careful combination of fluorescent lectin probes and confocal scanning laser microscopy (Lawrence et al., 2007, 2016; Aldeek et al., 2013). Microdomains are now realized to contribute to the smaller-scale heterogeneity that is observed within both suspended aggregate- and attached-biofilms. The presence of microdomains re-enforces the idea that the EPS matrix is not an amorphous, homogeneous entity, but rather can be structured at several different levels (Mayer et al., 1999; Decho, 2000a; Lawrence et al., 2003).

#### Proteins

Proteinacious moieties are common in natural EPS matrices and occur in a variety of molecular forms such as peptides, amino-sugars, glycoproteins, proteoglycans, and amyloid proteins (Gutierrez et al., 2007a,b; Fong and Yildiz, 2015; Zhang et al., 2015). They also can be grouped by their functions and properties such as extracellular enzymes (*e*-enzymes), membrane vesicle proteins, adhesins, amyloids, hydrophobins, and amphiphiles (Hobley et al., 2013). It is not well-understood yet, how proteins interact with other matrix molecules to accomplish these apparent functions.

While many proteins and peptides are easily hydrolyzed by microbial heterotrophy, certain proteins and peptides can be quite refractory to degradation. It is now realized that structured refractory complexes, called *amyloid* fibrils are a common component of EPS. These have not received much attention in oceanic environments but may contribute to a number of forms of refractory organic matter, and the refractory portions of EPS. Here, they may have important functions. Amyloids may form an important, refractory structural component of the EPS matrix (Gebbink et al., 2005; Larsen et al., 2007; Zheng et al., 2015).

Amyloids are loosely defined as any fibrillary polypeptide aggregate having a cross-β-quaternary structure (Fandrich, 2007), which self-assemble under the right environmental conditions. Amyloid fibrils consist of sets of 4–6 peptides linked together in a twisting, helical (i.e., rope-like) structure that is held together by non-covalent associations. A growing body of evidence supports the idea that amyloid fibrils, sometimes called curli fibers, may be formed from many different proteins (and peptides) and are a generic structure of peptide chain. While amyloid formation has been linked to many human disease processes (Barnhart and Chapman, 2006; van Gerven et al., 2015), they occur in natural microbial systems as a component of EPS. Their formation, at present, is thought to result from non-biological processes (Romero et al., 2010).

#### Extracellular *e-*DNA

A growing body of research now acknowledges the presence of extracellular forms of deoxyribonucleic acids (*e*DNA), and their role as an important structural component of the biofilm matrix (Böckelmann et al., 2005). Historically, *e*DNA was thought to result largely from the lysis of cells or release of plasmids. However, seminal studies by Whitchurch et al. (2002) showed the presence of *e*DNA as a part of the EPS. Concentrations of *e*DNA in sediments are often 3–4 orders of magnitude higher than those in the water-column, and suggest a role in the cycling of P in marine systems (Dell’Anno and Corinaldesi, 2004). Both *e*DNA and extracellular nucleases, together, may influence the physical consistency of biofilm EPS (Rice et al., 2007; Seper et al., 2011). Results of other studies indicated secretion by bacteria of *e*DNA is an active process (Nishimura et al., 2003; Steinberger and Holden, 2005; Suzuki et al., 2009; Gloag et al., 2013; Okshevsky and Meyer, 2013; Tang et al., 2013). The postulated roles suggest that *e*DNA may be a bacterial strategy that serves as an abundant structural scaffold within EPS. For example, in non-marine systems, the Gram-negative bacterium *Pseudomonas aeruginosa* uses *e*DNA bound to a specific cationic extracellular polysaccharide *pel* to provide structural stability to the EPS of biofilm (Jennings et al., 2015). Others have suggested an electron-transfer conduit, or substratum for the controlled movement of bound *e-*Enzymes (Flemming et al., 2007). Further studies await empirical testing of these ideas. However, an interesting caveat is that a recent study by Dell’Anno and Danovaro (2005) showed that DNA sorbed to sediments constituted an important source of phosphorus in normally P-limited deep-sea ecosystems. A review on *e*DNA pools in marine sediments summarizes many important aspects (Torti et al., 2015). Finally, DNA has been implicated in long-distance electron transfer processes (Giese, 2002). A pertinent question that warrants investigation is: does this offer the possibility for long-distance transfer of extracellular electrons through the EPS matrix?

#### Modifications Post-secretion

Extracellular polymeric substance, once-secreted by cells, are subjected to substantial environmental modifications, perhaps in predictable manners. Degradation of EPS may consist of a multi-step process. The steps likely represent the degradation of different components, ranging from highly labile to relatively refractory, which have different compositions and/or steric availabilities (to extracellular enzymes). A study of EPS produced within lithifying microbial mats showed that initial hydrolyses involved a rapid, and possibly selective, utilization by heterotrophs of certain sugar monomers, and LMW compounds. Certain components of the EPS, such as the uronic acids were highly labile to mat bacteria (Decho et al., 2005). Initial heterotrophic degradation of EPS was fueled by the large pool of LMW organics released by cyanobacteria during photosynthesis. This pool was consumed within 4–6 h post-daylight. The results indicated that a rapid, initial degradation occurred, followed by a much slower decomposition, leaving behind a more “*refractory remnant*” that persist for extended periods. As alluded to above, specific components of EPS such as many polysaccharides should exhibit relatively rapid turnover rates, when compared to more refractory components such as amyloid proteins. Finally, degradation will be affected by the physical properties of the EPS such as their gel versus solution states.

### Sorption, Trapping, and Diffusion-Slowing Properties

Diffusion is a key process in the design of the microbial cell, as it is the primary means by which small organic molecules and ions may be taken up by cells. Diffusion is also of relevance in the movement of signal molecules (i.e., autoinducers) involved in QS, extracellular enzymes, and antimicrobial agents. EPS can be broadly considered a sorptive sponge for the binding, trapping and concentration of organics and ions (Decho, 1990). Over the small spatial scales of biofilms, the EPS matrix influences the diffusion process. Diffusion is a multi-faceted process that is influenced by temperature, ionic concentrations, etc. A major driver in diffusion, of course, is the relative concentration gradient (Brogioli and Vailati, 2001). The density and properties of the EPS can influence diffusion rates (of ions or molecules) so they can range negligible (compared to diffusion in pure water at the same temperature) to having significantly slowed diffusivities (Decho, 2015).

Although bulk measurements of diffusivity may be estimated, it is difficult to determine how the smaller-scale variability in EPS densities influence diffusion at these scales. The natural matrix of EPS within aggregates or surface biofilms is often filled with channels, which by microbial design or by environmental influence, enhances mass transfer to/from cells.

A number of investigators have carefully measured diffusion rate constants by monitoring the movement of fluorescent molecules over time using confocal scanning laser microscopy and other approaches (Lawrence et al., 1994; Guiot et al., 2002; Stewart, 2002; De Beer et al., 2004; Waharte et al., 2010; Neu and Lawrence, 2014). Lawrence et al. (1994) initially used fluorescent molecules and confocal scanning laser microscopy to examine diffusivities and found them to be variable, ranging from those of pure water (*d* = 1.0) to extreme diffusion-slowing effects (*d* = 0.02) by the matrix. From a practical standpoint, one can assume there will be variability within an aggregate or attached biofilm. Collectively, these studies have shown that considerable changes occur in the movement of molecules and ions over microspatial distances (i.e., μms), which can relate to the observed heterogeneity that occurs within biofilms (Stewart and Franklin, 2008).

Physical trapping of organic and inorganic colloids, and nanoparticles also occurs in the EPS matrix. The viscoelastic nature and the dispersed arrangements of EPS at the surface-most fringes of biofilms make them ideal for the physical trapping of colloids, small particles, and/or sorption of ions and molecules. Sorption is influenced by a number of factors. These include pH, the forms and concentrations of ion(s), and the type(s) of ligands (binding sites) and associations (e.g., ionic- and covalent-bonds, van der Waals forces, etc.). The pH can have a strong effect on ionic binding, especially with regard to many EPS (Braissant et al., 2007, 2009; Gutierrez et al., 2008). In general, acidic pH tends to inhibit ion binding, while neutral or basic pH tend to promote binding. A caveat is that not all ions bind equally. A second caveat is that certain functional groups bind ions more efficiently at a given pH. For example, as the pH rises to near neutral, more complexation may occur to carboxyl sites. The increase in binding of divalent cations often results in a more cohesive polymeric gel structure.

This pH-dependent sorption process has practical importance in ocean systems (and biofilms) because the most abundant divalent ions in seawater are Ca^2+^ and Mg^2+^. These ions often form suitable (bi-dentate) bridges between adjacent EPS molecules having carboxylic acid groups, and can contribute to gel formation or floc (marine snow) formation in the water column. However, other important ions (at a given pH) may ‘outcompete’ Ca^2+^ and Mg^2+^ for binding sites. The binding of transition and other metals, such as Th, Cd, Cu, Ag, Fe, and Se, to EPS isolated from different environments, such as hydrothermal vents, microbial mats, and other areas, has been described (Schlekat et al., 1998; Zhang et al., 2008; Moppert et al., 2009; Deschatre et al., 2013). Metal binding to EPS of surface floc material (i.e., marine snow) in the surface waters of oceans, and subsequent sinking of flocs may result in significant vertical transport (flux) of trace elements to ocean floor, a process of global biogeochemical significance (as mentioned above).

While ocean seawater is often pH 7.8–8.2, the range of pH over smaller spatial (and temporal) scales can be quite dramatic. In microbial mat systems, where highly active bacterial respiration and photosynthesis occur, the pH has been shown to vary from pH 6.0–10.0 over a 24 h (i.e., diel) cycle (Visscher et al., 2000; Des Marais, 2003; Baumgartner et al., 2006). This is due to net photosynthesis during daylight (which raise pH), and net respiration during darkness (which lowers pH). This can result in regular diel changes in the complexation of ions, and hence influence the physical stability of EPS over a 24 h cycle. The diffusion-slowing properties of EPS contribute to the sharp geochemical gradients often observed within aggregate and attached biofilms (Baumgartner et al., 2006).

### Optical Properties

The biofilm is an organic gel coating (of EPS) on a surface (or within a suspended aggregate) with a collage of cells, and sorbed or localized molecules and ions, colloids, and particulates. All of these different components, individually or interactively, will influence the optical properties (i.e., refraction, scattering, and absorption of photons) of the broader surface (or water). The EPS can be thought of as a “semi-translucent” gel having different densities. Several processes act in concert to alter the optical properties of sediments. First, the polymers themselves appear to decrease the reflectance of the surface. In sediment systems, this can alter the amount of light entering the sediments. This is due to a combination of two processes. First, the gel polymers increase the spacing between sediment grains. This allows more light to enter in the spaces between grains, rather than being reflected from closely packed sediments. Second, the gel state of the polymer acts as a ‘photon trap’ because it mediates a change in refractive index, relative to seawater. This enhances the forward-scattering of photons, relative to back-scattering. This has been termed the ‘biofilm gel-effect’ (Decho et al., 2003). The functional value is that light may be more homogenously scattered around photosynthetic cells, and allow cells to conduct photosynthesis deeper in sediments (or mats).

As light interacts with a surface, photons are either reflected, scattered, refracted and/or absorbed. Reflectance involves back-scattering of photons at a fixed angle (relative to incident direction of photon). Often photons are ‘scattered’ at many angles relative to the incident. Refraction involves continuing through the surface, but altering the angle (relative to the incident) resulting in a change in refractive index. Absorbance involves the capture of photon energy by surface molecules or atoms. Absorbed photons may be re-emitted, as fluorescence (within pico-sec to nano-secs after absorbance), or released as heat. However, the biofilm is not simply a translucent gel but rather a three-dimensional matrix harboring cells, sorbed, or localized molecules (e.g., scytonemins, amino acids, etc.), colloids, and particulates. Biological chromophores (molecules that absorb light near specific wavelengths) include the purines and pyrimidines of DNA, the ‘ringed’ amino acids (tyrosine, phenylalanine, etc.), and other molecules. The sea surface layer is known to harbor EPS gels (Wurl and Holmes, 2008). Of special interest will be how the sea-surface layers of EPS influence photon penetration into the underlying water.

## Localization of Microbial Extracellular Processes

### Quorum Sensing

Do microbial communities communicate and coordinate activities? Classical microbiology during much of the past century has taught us to understand microbes simply as individual cells. Recently, however, a growing body of evidence supports the idea that bacteria often act in groups, rather than as individuals. When bacteria are attached, their proximity to each other results in the development of interactive relationships ranging from antagonistic to agonistic, and even altruistic. These interactions are often chemically mediated but are tempered by the ever-changing conditions of their local environment. The diffusion-slowing properties of the EPS matrix facilitates the development of such relationships among cells in a way that cannot be accomplished by free-living planktonic cells.

Quorum sensing is a type of bacterial cell–cell communication that involves the exchange of chemical signals among nearby cells to coordinate behaviors that are best conducted in groups (Fuqua et al., 1996). It involves the production, detection, and response by cells to diffusible signaling molecules (i.e., autoinducers). Autoinducers accumulate in the proximal environment as the bacterial population increases. When autoinducer concentration reaches a threshold-level, cells collectively alter gene expression. Many group activities such as bioluminescence, antibiotic production, and EPS secretion (Camilli and Bassler, 2006) are regulated by QS.

Quorum sensing can also be utilized by cells for ‘*diffusion-sensing’* (Redfield, 2002). This allows bacteria to sense the diffusional properties of its proximal environment, presumably to ‘make decisions’ whether to conduct more metabolically costly processes, such as production and release of extracellular enzymes, plasmids, antibiotics, etc. (Ruparell et al., 2016). Together, these two processes, quorum- and diffusion-sensing, have been termed ‘*efficiency sensing*’ (Hense et al., 2007).

The foundation for cell–cell cooperative interactions originally was proposed for explaining the bioluminescence by a marine luminescent bacterium, previously *Photobacterium*, renamed *Vibrio fischeri*, and then *Aliivibrio fischeri*, which was isolated from a small Hawaiian squid (Ruby and Nealson, 1977; Nyholm et al., 2000). Studies progressively showed that autoinducer molecules, upon reaching a threshold concentration in the medium, triggered changes in gene expression that resulted in bacterial luminescence. Luminescence by populations of symbiotic bacteria, localized in the light organs of the squid, afforded it a selective advantage against predation. The ability to communicate, coordinate, and act as groups, however, does not relinquish the cells as an individual unit. Microbial cells can (and do) still act as individual cells. This amazing flexibility likely contributes to the tremendous success and resiliency of bacteria.

In open surface-water ocean environments, QS can have large-scale effects, especially when in overwhelming abundances. An obvious example of this was the ‘milky ocean’ that was observed at night by satellite off of Somalia, Africa (Nealson and Hastings, 2004; Miller et al., 2005). The milky ocean, which was 100s of square km in size, was due to QS-triggered bioluminescence in ocean surface populations of bacteria.

Several different classes of chemical signals exist. Most were described from the study of infection-causing bacteria. These include acylhomoserine lactones (AHL), unique oligopeptides, furanosyl borate diesters (Autoinducer-2), and gamma-butyrolactones (Waters and Bassler, 2005). The AHLs comprise a class of approximately 18 different types of signal molecules that are released into the surrounding environment, and eventually bind to an intracellular receptor protein, whose complex then triggers changes in gene expression (Churchill and Chen, 2011). AHLs in marine environments have been found in sponges (Taylor et al., 2004), microbial mats (McLean et al., 1997; Decho et al., 2009, 2010) and marine snow (Hmelo et al., 2011). Interestingly, signals such as AHLs are prone to inactivation under certain environmental conditions such as high pH (>8.0) (Decho et al., 2009; Hmelo and van Mooy, 2009). It is not known how fluctuating conditions (e.g., pH, oxidants, desiccation, photocatalytic degradation) in natural environments influence chemical signaling and coordination of microbial activities (Horswill et al., 2007; Decho et al., 2009, 2011; Frey et al., 2010). Signaling, however, will likely be localized and most pronounced within EPS matrices, and within planktonic aggregates (Gram et al., 2002; Wagner-Dobler et al., 2005; Hmelo et al., 2011; Amin et al., 2015; Jatt et al., 2015).

Signaling using AHLs, for example, is not limited to heterotrophic bacteria. Rather it has been found in photosynthetic cyanobacteria (Sharif et al., 2008) and Archaea (Zhang et al., 2012). Importantly, it is now realized that this form of cell–cell communication can occur in single-species population, but may also be utilized by inter-Kingdom consortia, such as plant-microbe and animal–microbe associations. Contrastingly, molecules that may act as signals for some bacteria, may act as antibiotics against other bacteria (Kaufmann et al., 2005; Davies et al., 2006; Schertzer et al., 2009; Johnson et al., 2016). A final note is that currently only several classes of signaling molecules (e.g., AHLs, AI-2, and peptides, diffusible signal factors (DSFs), etc.) are known (Schaefer et al., 2008; Papenfort and Bassler, 2016, for review). However, QS and similar interactions via chemical signaling are likely to occur using a variety of signals; most of which may be unknown at present.

### Extracellular Vesicles and Gene-Exchange

Bacteria possess the capability to bud-off portions of their cell membranes (Schooling et al., 2009; Biller et al., 2014), which are then released as extracellular vesicles. The vesicles provide bacteria with the ability to package molecules within a surrounding lipid membrane, and release them in their surrounding extracellular environment, and localize them within the EPS matrix. This can provide a protective ‘minefield’ against antibiotics, preserve extracellular signals and plasmids, and provide other functions as well. The presence of extracellular vesicles within biofilms, and specifically the EPS matrix, is now realized to be quite common (Mashburn and Whiteley, 2005; Biller et al., 2014). The vesicle composition is often similar to the plasma membrane (Gram-positives) or outer cell membrane (in Gram-negatives), but additionally contain specific proteins as part of the vesicle. The vesicles can package a wide range of molecules such as *e*DNA, RNA, *e*-enzymes, antibiotics, and signal molecules, and likely provide protective effects for the packaged molecules they carry (Mashburn-Warren et al., 2008; Schooling et al., 2009).

Gene exchange is an important process among bacteria. The EPS matrix can enhance gene exchange among cells for several reasons. First, conjugation (i.e., a uni-directional exchange of plasmids via a pilus connecting two cells) requires extended contact for a prolonged period of time (approximately 20 min). In open-water systems, this is difficult due to Brownian motion constraints. When localized in a three-dimensional EPS matrix, two cells can remain relatively stationary for prolonged periods of time, which can facilitate conjugative gene exchange. Second, extracellular DNA, used in transformation, can be rapidly degraded once outside of the cell, or strongly sorbed to sediment particles. When DNA is immobilized within EPS, its persistence can be enhanced, and thus increase chances for transformational exchange of DNA among cells. Direct measurements of these two processes, to our knowledge, are not yet available.

### Extracellular Enzymes (e-Enzymes) and Hydrolysis products

Degradation of organic matter and its mineralization to CO_2_ is a fundamental process of bacteria. In many ocean environments, bacteria produce *e*-enzymes to partially hydrolyze organic matter that becomes sorbed or trapped by the EPS (Hoppe et al., 2001). When conducted efficiently, with minimal loss to the surrounding water, the biofilm can be an efficient external digestion system for the microbial community (Flemming and Wingender, 2010).

The localization of *e*-enzymes is a process that is important in open water aggregate- as well as attached-biofilms. In order for efficient diffusional uptake, both enzymes and their hydrolysis products must remain localized in proximity to cells (e.g., approximately 30 μm). EPS provide a matrix to localize both enzymes and their hydrolysis products relatively close to cells. It is not known, however, how *e*-enzymes remain localized within the EPS matrix. Are they attached (bonded) to polymers with active sites exposed? In studies of other systems, bacteria are known to localize polysaccharases and other *e*-enzymes (Sutherland, 1999, 2016). *e*-Enzymes are also known to be contained within extracellular vesicles, localized within the expolymer matrix (Mashburn and Whiteley, 2005; Mashburn-Warren et al., 2008; Elhanawy et al., 2014), and e-DNA nucleases were found in *Vibrio cholerae* biofilms (Seper et al., 2011). Indeed, elevated microbial activities, such as enzymatic activities, have been reported in marine snow particles at higher-levels than those in surrounding sea water (Smith et al., 1992; Ploug et al., 1999; Grossart et al., 2003; Jatt et al., 2015), thus suggesting that marine snow are hotspots for remineralization of organic and inorganic materials (Azam and Long, 2001; Thornton et al., 2010).

Insight has been provided through studies of other systems (Tielen et al., 2013). They showed that extracellular lipase was protected against heat denaturation via complexation with the EPS alginate. Using molecular modeling they were able to show that *e*-enzymes can be physically bound to EPS, however, the enzyme/EPS bond must occur away from active sites on the enzyme. Finally, this bonding provides enhanced stability against denaturation. However, questions remain, such as: how are enzymatic activities maintained outside the cell? Empirical evidence has been relatively limited. Do the functional equivalents of extracellular chaperones help to maintain activities (i.e., prevent denaturation) of *e*-enzymes? It is also not known how extracellular enzymes may modify the EPS themselves.

## EPS in Microbial Mats and Mineral Precipitation

Microbialites are benthic microbial deposits (Burne and Moore, 1987). Microbial mats, a type of microbialite, are the longest-lived ecosystems that are known to have existed on Earth. Certain fossilized microbialites extend far back in the fossil record (Sprachta et al., 2001; for review, see Chagas et al., 2016). They are the earliest known macro-fossil evidence of life in the geological record, extending back an estimated 3.4–3.7 gy (Tice and Lowe, 2004; Nutman et al., 2016). They dominate the fossil record for 3 gy, which represents over 80% of the time life has existed on Earth (Allwood et al., 2007). Recently, the precipitation process has been studied at nanometer spatial scales (Benzerara et al., 2006).

Microbial mats typically exhibit a distinct vertical layering of microbial functional groups that is strongly influenced by externally influenced gradients such as light and geochemical conditions (Des Marais, 2003; Vasconcelos et al., 2006; Franks and Stolz, 2009). In most cases, mats are examples of actively metabolizing, highly organized microbial communities, and constitute “high-yield” systems where resources are efficiently recycled amongst its members (Visscher and Stolz, 2005). These systems, therefore, offer excellent platforms from which to study how EPS may influence the precipitation of carbonate minerals. Thrombolites (Mobberley et al., 2015) and tufa deposits (Zippel and Neu, 2011; Dupraz et al., 2013) are other forms of microbialites.

Extracellular polymeric substances are abundantly present in microbialites, such as mats and contribute to the metabolic efficiency of mat communities (Neu, 1994). This occurs through their diffusion-slowing properties, light-attenuation, and abilities to influence 3D-architecture, chemical communication, extracellular enzymatic hydrolyses, and biogeochemical mineral precipitation. The details of how this relate to the molecular-scale interaction occurring between ions and the EPS are not, as yet, fully understood.

### Carbonate Precipitation

Mats are well-known for their association with the biogeochemical precipitation of minerals such as carbonates (e.g., calcite, aragonite) (Decho, 2010). Present-day examples of precipitating mats include tufa mats, marine stromatolites, and marine thrombolites (see Dupraz et al., 2009, for review; Glunk et al., 2009; Tourney and Ngwenya, 2014). There are several different mechanisms known to directly or indirectly influence precipitation within mat environments (Visscher and Stolz, 2005). Microbial communities drive the basic alkalinity engine, which when coupled to the organic matrix of mats, results in biogeomineral precipitation (Dupraz and Visscher, 2005). Activities of several microbial groups, such as cyanobacteria, sulfate reducers, and anoxygenic phototrophs, can ‘*promote precipitation*,’ while other groups (e.g., aerobic heterotrophs, sulfur oxidizers, and fermenters) can ‘*promote dissolution*’ (Dupraz et al., 2009). Precipitation of CaCO_3_ occurs in seawater that is near or exceeding supersaturation of carbonate ions, and has basic pH conditions (Arp et al., 2001, 2003). Cyanobacterial activities, for example, will raise the pH during daylight photosynthesis, which favors localized carbonate precipitation (Gautret et al., 2004; Ludwig et al., 2005). Specific moieties on EPS, such as acidic groups, can act as nuclei for subsequent CaCO_3_ precipitation (Braissant et al., 2003; Bhaskar and Bhosle, 2005; Obst et al., 2009). Even bacterial cells themselves can serve as nucleation sites for precipitation (Varenyam et al., 2010). EPS can bind substantial amounts of free Ca^2+^ (Braissant et al., 2007) and other minerals such as phosphate and sulfate (Gallagher et al., 2013) from the surrounding water. This can result in precipitation of EPS-associated minerals such as apatite [Ca_5_(PO_4_)_3_(F,Cl,OH)], struvite (MgNH_4_PO_4_⋅6H_2_O), dolomite [CaMg(CO_3_)_2_], and aragonite (Gallagher et al., 2012, 2013).

Under some conditions, however, EPS can inhibit precipitation, or even contribute to carbonate dissolution. Cation-binding by EPS removes free Ca^2+^ ions from solution, through depletion of carbonate minerals from the proximal surroundings. Acidic amino acids, such as aspartic or glutamic acids, and carboxylated polysaccharides (i.e., ${\mathrm{CO}}_{3}^{2-}$ groups of uronic acids) can act as strong inhibitors of CaCO_3_ precipitation (Kawaguchi and Decho, 2002a,b; Dupraz et al., 2004; Gautret and Trichet, 2005). The functional groups and their steric availability (to bind ions) are key in this process (Rieger et al., 2007; Yang et al., 2008). The role(s) in carbonate precipitation of specific microbial clades such as sulfate reducers, however, remains controversial (Meister, 2013), but likely involves different types of community interactions under normal marine, hypersaline, and alkaline conditions (Gallagher et al., 2014).

Marine stromatolites are microbial mats having repeating layers of precipitated micritic laminae produced through interaction of the microbial communities and the environment (Krumbein, 1983). In present day, they occur in only a few limited marine environments such as the Bahamas (Reid et al., 2000; Paerl et al., 2001) and Shark Bay in Western Australia (Goh et al., 2009). Studies extending for over a decade have examined present-day open-water, subtidal, marine stromatolites at Highborne Cay (Bahamas) and showed the surface microbial community consisted of several distinct mat stages (i.e., termed Types 1, 2, and 3), each having very different phenotypic characteristics (Reid et al., 2000). The EPS produced during these stages had very different properties and influenced the microbial communities within. In the Type 1 stage, the community exhibited “high growth.” It consisted of dense cyanobacteria with high EPS production that grew (upward) quickly, consuming resources (Decho et al., 2005). The abundant EPS resulted in a “sticky” surface that trapped ooid grains (i.e., sediment) washing over the mats during high wave actions; a process that propagated the continued upward growth of the mat. The EPS contained ligands to chelate much of the available free Ca^2+^ ions. The net result was that EPS in the Type 1 mat inhibited CaCO_3_ precipitation (Visscher et al., 2000). When a Type 1 mat transitions to a Type 2 mat, sulfate-reducing bacteria (SRB) increase in their relative abundances. EPS, initially produced by the cyanobacteria, are consumed by SRBs, then re-secreted as different EPS, a process which enhances localized precipitation (Visscher et al., 2000; Decho et al., 2005). Infared (FT-IR) spectral analyses of EPS extracted from the precipitate closely resembles those extracted from SRB mat isolates (Braissant et al., 2009). Notably, the EPS properties change in the Type 2 mat, becoming “less sticky.” These studies illustrated how the EPS properties of the different mat could influence their properties and growth. The Type 2 mat resembles a classic microbial mat, with less EPS production (or accumulation), and little/no upward growth. The cyanobacteria, SRB, sulfur-oxidizing bacteria (SOB), and aerobic heterotrophs become spatially organized, and exhibit a closer metabolically coupling.

With rising levels of atmospheric CO_2_, efforts are beginning to examine if certain carbonate-precipitating bacteria can be used to sequester (and store) CO_2_ (Paul et al., 2017) and understand how microbial mat processes influence C storage (Bouton et al., 2016).

### Sediment Stabilization and Fossil Evidence of Mats

Sediment fluxes in marine systems are affected by many parameters, including sediment grain sizes, physically cohesive muds, and biologically cohesive microbial extracellular polymers (Gerbersdorf et al., 2009; Grabowski et al., 2011). EPS concentrations in marine sediments vary considerably (Underwood et al., 1995, 2004). EPS, especially those from microphytobenthos (e.g., diatomaceous mats), are important in cohesive sediment stability, and resistance against erosion and resuspension (Grant and Gust, 1987; Paterson, 1989; Smith and Underwood, 1998, 2000; Tolhurst et al., 2002; Underwood and Paterson, 2003; Hanlon et al., 2006). Levels of EPS present in sediments can be a crucial variable to sediment stability (Malarkey et al., 2015). However, more EPS isn’t always better. Interesting experimental studies by Paterson et al. (2008) showed that lower levels of EPS were more efficient in increasing erosional thresholds than abundant EPS conditions. While is well-established that the cohesive properties of EPS contribute to sediment stability, it is however not well-understood how the molecular-scale interactions of EPS themselves contribute to their cohesiveness (Paterson et al., 2008). Sediment-inhabiting small animals may indirectly influence sediment stability. For example, increased EPS production was shown to occur in the presence of a grazing nematode (Hubas et al., 2010). Initial studies suggest that QS (see above) may be involved in biofilm formation in certain diatoms (Yang et al., 2016) and offers the possibility that QS may contribute to the sediment stabilization process.

Finally, the very same bedform patterns that contribute to sediment stability in present-day sediments (e.g., ripples) also are considered as fossil evidences of sediment stabilization. These include patterns such as microbially induced sedimentary structures (MISSs), which are considered to be indirect fossil remnants that illustrate the very earliest vestiges of microbial mat life through geologic time (Noffke et al., 2001, 2013; Noffke, 2010).

## Animal–microbial Interactions and Food-webs

### Feeding Studies

It was realized early on that biofilms, and more-specifically their EPS, can represent a potentially labile carbon source for animals ingesting microbial cells (Decho, 1990). Since many small animals, present in both the water column and sediments of ocean systems, ingest microbial flora as a food source, they will coincidently ingest the closely associated EPS during the feeding process. Many invertebrate taxa are filter-feeders (i.e., straining suspended particles from the water) or deposit-feeders (i.e., ingesting sediments and their organics), therefore will consume microbial flora and their associated EPS as a food. Initial feeding experiments addressing EPS utilization by marine animals were conducted using EPS that were isolated from bacterial cultures grown in the presence of radioactively labeled (^14^C) substrates. Once the EPS were separated from cells and residual label, EPS were mixed with sediments and fed to animals. Results indicated that EPS comprised a highly labile carbon food source. Examples of such studies involved copepods (Decho and Moriarty, 1990), polychaete worms (Decho and Lopez, 1993), bivalves (Harvey and Luoma, 1985), and sea stars (Hoskins et al., 2003). Biofilms are even known to be grazed upon by benthic foraminifera (Bernhard and Bowser, 1992).

Extracellular polymeric substance can act as a ‘sorptive sponge.’ This is due to their ability to bind metals, other ions, and even relatively hydrophobic organic contaminants such as pesticides, which is attributed to the presence of charged moieties (i.e., positive or negatively charged functional groups) or hydrophobic moieties. This can result in the efficient trophic-transfer of metals and pesticides to consumer animals that are ingesting EPS. Together, these moieties serve to sorb and/or trap, and concentrate environmental contaminants. When animals ingest the matrix, coincidentally during their feeding on sediments, cells or flocs, they will consume the sorbed metals and organics as well. EPS have been shown to be an efficient trophic-transfer vehicle for sorbed metals in amphipods (Schlekat et al., 1998, 1999, 2000; Selck et al., 1999), and organic compounds such as pesticides, although most of the latter work has even been conducted in freshwater systems (Widenfalk et al., 2008; Lundqvist et al., 2010, 2012).

#### Binding of Nanoparticles to EPS and Biofilms

In ocean systems, both natural (e.g., geological) processes and anthropogenic processes result in the generation of extremely small particulates called nanoparticles. Nanoparticles measure 1–100 nm in at least one dimension, and have different physicochemical properties than larger particles. In oceans, nanoparticles occur in the form of metal contaminants, organics, and even degraded plastics and potentially may have long water-column residence times owing to their small sizes. A number of studies are indicating that nanoparticles are efficiently concentrated by biofilms, and more specifically their EPS (Battin et al., 2009; Ferry et al., 2009; Fabrega et al., 2011; Nevius et al., 2012; Ikuma et al., 2014, 2015). Further studies have shown that nanoparticles are taken up by protozoans (Holbrook et al., 2008; Werlin et al., 2011; Mortimer et al., 2016). Finally, the bioavailability and bioaccumulation of nanoparticles occurs in marine (and freshwater) animals such as snails, bivalves and oligochaetes (for review, see Luoma et al., 2014), all of which coincidently ingest EPS during feeding processes. Therefore, EPS can work as a trophic transfer vehicle for nanoparticles to enter food webs.

#### EPS Capsules, Survival of Digestion, and the Gut Microbiome

Bacteria often possess EPS capsules surrounding their cells. The presence of capsules is a protective measure for the cells. Studies conducted by Plante and colleagues showed that encapsulated bacteria were less susceptible to digestion during detritivore- and deposit-feeding (Plante et al., 1990; Plante and Schriver, 1998; Plante, 2000). DePas et al. (2014) showed that protection is afforded to biofilm bacteria during grazing by the nematode *Caenorhabditis elegans*.

Finally, one understudied aspect of trophic interactions regarding biofilms that is gaining attention involves the roles of resident gut bacteria in consumer animals (including humans). The presence of these bacteria, whose densities and activities may be quite substantial, is often facilitated by EPS capsules and biofilms. Gut bacteria are a source of many new genes, and the diversity and ecological principles driving these microbiomes will be an interesting future area of study (Dorosz et al., 2016).

### The Larval Settlement Process and Biofouling

Virtually any type of surface, when placed in seawater becomes fouled with organisms ranging from bacteria to animals and algae; a process known as biofouling (Lewin, 1984). Bacteria and other microbes are generally the initial colonizing organisms of a surface. The presence of a bacterial biofilm often sets the stage for subsequent larval settlement (Tran and Hadfield, 2011). Understanding how biofilms interact with larval settlement is important to the broader biofouling process, which constitutes a costly ocean-engineering problem.

Studies in marine systems suggest that larval settlement is also a multistep process, which involves initial sensing of specific chemical cues, initial settlement and “tasting” of the surface, and finally more-permanent settlement. The initial sensing step of waterborne cues is a concentration-dependent process.

Presently, data suggest that in many cases, the larval settlement cues are molecules that are produced by adult conspecifics and are concentrated within the surface biofilm matrix or, in some cases, may be produced by the biofilms themselves (Unabia and Hadfield, 1999; Bao et al., 2007). Cue(s) can be multifunctional, acting as agonists, antagonists, or toxins (Ferrer and Zimmer, 2012; Guezennec et al., 2012). Behaviors and responses of larvae to cues are often species-dependent.

Settlement cues may be localized within the biofilm, and more-specifically by the EPS matrix. Diatom biofilms and the possible involvement of heat-stable settlement cues are involved in the settlement of the polychaete *Hydroides elegans* (Lam et al., 2003). *Hydroides* sp. are examples of the initial colonizers of open surfaces in warmer water regions. Once they are set, the complexity of colonizing species increases. Settlement cues may be produced by the biofilm itself but has been challenging to verify. The beneficial effect of a specific epibiotic bacterial biofilm on marine animal or plant hosts has been suggested (Holmström et al., 1992; Tran and Hadfield, 2011). Coralline red algae, for example, are highly inductive surfaces for the settlement of marine invertebrates, and are now realized to be strongly influenced by the surface microbial flora and their cues (Nielsen et al., 2015).

In contrast, the inhibition of larval settlement seems to be influenced by waterborne or biofilm-associated molecules. This is important to the potential control of biofouling. Early studies noted that many marine animals and macroalgae exhibit reduced biofouling, and suggested that chemical defenses may be involved (Holmström et al., 2002; Rao et al., 2007). More recently, studies have shown that specific chemical inhibitors can be produced by either the host organism (e.g., algae, animal) or by biofilm bacteria growing on the surface of the host (Lau and Qian, 2000). These have included both large and small molecules. In tunicates, proteins produced by biofilm bacteria inhibit further colonization by bacteria (Holmström et al., 1992). In a series of landmark studies, de Nys et al. (2009) showed the macroalga *Delissia pulchra*, was not subject to biofouling. This inhibition (of biofouling) operates by jamming the cell–cell chemical communication pathways of bacteria. As mentioned above, many gram-negative bacteria use AHLs in chemical communication. Release of specific AHL analogs, which are similar in molecular design can “jam” the QS pathway(s) of AHLs. Small halogenated furanones, resembling AHLs, interfered with chemical signaling in bacteria. An ecological function of antifouling molecules produced by plants and animals was postulated (and was tested) by Kjelleberg and colleagues (Franks et al., 2006). Harder et al. (2002) found large (>100 kDa) polysaccharide-containing molecules, produced by both a host macroalga (*Ulva* sp.) and *Vibrio* sp. bacteria, to inhibit larval settlement. These molecules act as a broad-spectrum inhibitor for settlement. This has touched off substantial exploration for chemically based inhibitors of biofouling in both nature and medicine by many laboratories. This infers the complex interaction in biofouling among host organisms, bacterial biofilms, and chemical cues. Since the biofouling of marine surfaces has both positive and negative effects to hatcheries, this area has an emerging impact on aquaculture processes (Joyce and Utting, 2015; Camacho-Chab et al., 2016). Finally, work is in progress to understand how climate change may affect processes such as larval settlement (Whalan and Webster, 2014).

## Extreme Ocean Environments

In extreme and fluctuating conditions, microbes surround themselves with EPS in an effort to add stability to their extracellular environment. The physiological plasticity of microbial cells, combined with their EPS-based adaptations allow microbial life to succeed at the boundaries of where other forms of life can survive.

### Low-temperature Sea-ice Communities

In polar regions, metabolic processes are slowed by relatively cold temperatures. It is here that microbes also employ EPS to their advantage. Earlier, pioneering studies in Antarctic systems showed the presence of specific ‘anti-freeze proteins’ (i.e., glycoproteins) within the blood plasma of fish (Devries, 1971). The proteins would bind to ice crystals as they formed and prevent further growth of damaging ice crystals in the blood. This realization launched many subsequent studies of other organisms, including bacteria.

In Arctic and Antarctic systems, the presence of EPS play key roles as cryoprotectants, for attachment to sea-ice interfaces, and to survive enclosure in ice (Underwood et al., 2010, 2013). Studies of bacterial isolates from sea-ice systems have demonstrated that certain glycoproteins and exopolysaccharides act as cryoprotectants, which inhibit ice crystal nucleation, in addition to securing the attachment of cells to the ice surface (Nichols C. A. et al., 2005; Nichols C. M. et al., 2005; Marx et al., 2009; Ewert and Deming, 2014). EPS and TEP become routinely embedded in sea-ice (Meiners et al., 2003; Collins et al., 2008) and contribute to the survival of microbial cells in these environments (Krembs et al., 2002, 2011; Liu et al., 2013; Boetius et al., 2015). EPS, including TEP can account for the majority of the carbon pool in sea-ice, which is later released during melting (Miller et al., 2011; Wurl et al., 2011), and can even make their way into atmospheric ice (Wilson et al., 2015).

### High-temperature Hydrothermal Vents

Since their initial discovery in 1977, ocean hydrothermal vent systems have received much scientific attention. They are located near specific regions of the ocean spreading centers of tectonic plates, where geothermally heated fluids, enriched in minerals, hydrogen sulfide, ammonia and methane are released and mix with much colder surrounding seawater. Mineral deposits form as chimneys, and are surrounded by islands of intense biological activity, where chemosynthetic bacteria and archaea form the base of a food web having a diversity of often unique animals (Tunnicliffe, 1991). For these reasons, they have been considered as a possible site for the origin of life on Earth, and as an analog for study in the exploration for possible life elsewhere.

Isolates of bacteria from vent systems demonstrate the capacity for abundant EPS production, perhaps to sequester dissolved minerals and other metals from the surrounding water (Raguénès et al., 1997a,b; Guezennec et al., 1998; Rougeaux et al., 1999, 2001; Guezennec, 2002). EPS, derived from isolate cultures, typically have uronic acid contents as high as 40%, and relatively high molecular masses (Guezennec, 2002). It is not yet understood, however, how the EPS may influence the microenvironment of the bacteria and archaea, in terms of *e*-enzymes, 3D-microspatial development of their communities, and microspatial acidification (to solubilize metal ions). It is not known if the EPS matrix facilitates these processes, and actually may serve to inhibit their precipitation, similarly to those in some shallow-water carbonate environments?

### Hypersaline Environments and Desiccation

Hypersaline systems, such as salt ponds, salterns, and hypersaline lagoons, contain well-developed microbial mats. Many of these systems occur in proximity or directly connected to ocean systems, while others are inland. Examples of hypersaline systems are numerous and a few include Salt Pond, San Salvador, Bahamas (Pinckney and Paerl, 1997); Guerrero Negro, Baha California Sur, Mexico (Ley et al., 2006); Laguna Tebenquiche, Salar de Atama, Chile (Fernandez et al., 2016); Don Juan Pond [McMurdo Dry Valleys, Antarctica (Dickson et al., 2013)]; Dead Sea (Oren, 1994); Solar Lake, Sinai, Egypt (Teske et al., 1998); Hamelin Pool, Shark Bay, Western Australia (Goh et al., 2009); Polynesian islands (Rougeaux et al., 2001; Richert et al., 2005; Moppert et al., 2009).

The hypersaline environment presents unique challenges to microorganisms, especially in terms of fluctuations in ion concentrations and osmolarity. In addition, many hypersaline mats are exposed to intermittent and/or progressive desiccation. A lack of available water, during the desiccation process, can kill a bacterial cell, largely through denaturation of proteins and destabilization of cell membranes (see Potts, 1994, for review). EPS are an abundant component of such mats (Benninghoff et al., 2016), and likely provide a degree of protection to mat microbial flora against ion fluctuations and desiccation (Potts, 1994; Shaw et al., 2003; Decho, 2016). Interestingly, studies have shown that bacteria can survive in a desiccated state in salt crystal for 250 my (Vreeland et al., 2000).

In hypersaline mats, EPS occur in the form of capsules surrounding individual cells, or a larger EPS matrix surrounding many cells in a biofilm, which can buffer cells against either dessication or rapid changes in water potential. Salinities in hypersaline ponds are often >300 g/L (e.g., seawater is approximately 32 g/L), but can reach as high as 440 g/L, often with concentrations of individual ions not matching those observed for typical seawater. Here, mat communities often experience extended periods (i.e., days to months) of desiccation that often is followed by a rapid rehydration due to seasonal rain events.

Selective saltation also plays a role in the ability of mats to cope with increasing salinities. This allows less-soluble minerals to be removed from solution, as a function of concentration. The evaporation process that occurs throughout the dry season serves to increase ionic concentrations and promotes the selective precipitation of salts on the mat surface. As ionic concentrations increase, there is a sequential salting-out occurring of less-soluble minerals. For example, much of the Ca^2+^ is typically removed as gypsum (CaSO_4_ 2H_2_O) or smaller amounts of calcite (CaCO_3_). Gypsum begins to precipitate when salinity concentrations reach about 160 ppt. At very high ionic concentrations (>300 ppt) NaCl begins to precipitate.

Some EPS may condense with increasing salinity, and even form a hydrophobic barrier on the surface of the biofilm. This may result in enhanced protection during subsequent desiccation. It has been proposed that the exclusion of ions occurs via the EPS matrix in response to increasing salinity, which is designed to reduce osmotic stress and conserve water within the mat (Decho, 2016). Desiccation is a process occurring in many areas of marine environments. On the fringes of ocean systems, specifically on the upper reaches of rocky intertidal zones, intermittent desiccation is a common process. The roles of EPS in stabilizing microbial communities require further investigation.

## Summary: EPS Research Looking Forward

The growing awareness of microbial EPS and their influences on ocean processes are evidenced in this special issue and offers many avenues for future research. It is emphasized here that the secretion of EPS is an adaptive response employed by microbes to enhance their metabolic efficiency and survival. An extensive literature on EPS and biofilms that is available in other areas of microbiology may have relevance to ocean studies.

Finally, there were many aspects of EPS that were not covered in this relatively short overview, but are important to understanding the dynamics of microbial extracellular biology. For example, we have not addressed: (1) EPS as electron-transfer vehicles; (2) the concentration of viruses; (3) molecular pathways of EPS secretion; and (4) the roles of biofilms in the search for life elsewhere. In addition, we anticipate that the roles of EPS in ocean systems will be integrated into the fundamental microbiology of the ocean, and into larger-scale topics such as global climate change, biotechnological applications of EPS, and the search for novel antibiotics and other medicinal compounds.

## Author Contributions

All authors listed, have made substantial, direct and intellectual contribution to the work, and approved it for publication.

## Conflict of Interest Statement

The authors declare that the research was conducted in the absence of any commercial or financial relationships that could be construed as a potential conflict of interest.

## References

[B1] Aldeek F. Schneider R. Fontaine-Aupart M. P. Mustin C. Lécart S. Merlin C. (2013). Patterned hydrophobic domains in the exopolymer matrix of *Shewanella oneidensis* MR-1 biofilms. *Appl. Environ. Microbiol.* 79 1400–1402. 10.1128/AEM.03054-12 23220957 PMC3568607

[B2] Alldredge A. L. Cohen Y. (1987). Can microscale chemical patches persist in the sea? Microelectrode study of marine snow, fecal pellets. *Science* 235 689–691. 10.1126/science.235.4789.689 17833630

[B3] Alldredge A. L. Cole J. J. Caron D. A. (1986). Production of heterotrophic bacteria inhabiting macroscopic organic aggregates (marine snow) from surface waters. *Limnol. Oceanogr.* 31 68–78. 10.4319/lo.1986.31.1.0068

[B4] Alldredge A. L. Passow U. Logan B. E. (1993). The abundance and significance of a class of large, transparent organic particles in the ocean. *Deep Sea Res.* 40 1131–1140. 10.1016/0967-0637(93)90129-Q

[B5] Alldredge A. L. Silver M. W. (1988). Characteristics, dynamics and significance of marine snow. *Prog. Oceanogr.* 20 41–82. 10.1016/0079-6611(88)90053-5

[B6] Allwood A. C. Walter M. R. Burch I. W. Kamber B. S. (2007). 3.43 billion-year-old stromatolite reef from the Pilbara Craton of Western Australia: ecosystem-scale insights to early life on Earth. *Precambr. Res.* 158 198–227. 10.1016/j.precamres.2007.04.013

[B7] Aluwihare L. I. Repeta D. J. Chen R. F. (1997). A major biopolymeric component of dissolved organic carbon in surface seawater. *Nature* 387 166–169. 10.1038/387166a0

[B8] Amin S. A. Hmelo L. R. van Tol H. M. Durham B. P. Carlson L. T. Heal K. R. (2015). Interaction and signaling between a cosmopolitan phytoplankton and associated bacteria. *Nature* 522 98–101. 10.1038/nature14488 26017307

[B9] Amon R. M. W. Benner R. (1994). Rapid cycling of high-molecular-weight dissolved organic matter in the ocean. *Nature* 369 549–552. 10.1038/369549a0

[B10] Amon R. M. W. Benner R. (1996). Bacterial utilization of different size classes of dissolved organic matter. *Limnol. Oceanogr.* 41 41–51. 10.4319/lo.1996.41.1.0041

[B11] Anton J. Meseguer I. Rodriguezvalera F. (1988). Production of an extracellular polysaccharide by *Haloferax-mediterranei*. *Appl. Environ. Microbiol.* 54 2381–2386. 16347749 10.1128/aem.54.10.2381-2386.1988 PMC204266

[B12] Arnosti C. Ziervogel K. Yang T. Teske A. (2015). Oil-derived marine aggregates – hot spots of polysaccharide degradation by specialized bacterial communities. *Deep Sea Res. II* 129 179–186. 10.1016/j.dsr2.2014.12.008

[B13] Arp G. Reimer A. Reitner J. (2001). Photosynthesis-induced biofilm calcification and calcium concentration in Phanerozoic oceans. *Science* 292 1701–1704. 10.1126/science.1057204 11387471

[B14] Arp G. Reimer A. Reitner J. (2003). Microbialite formation in seawater of increased alkalinity, Satonda Crater Lake, Indonesia. *J. Sed. Res.* 73 105–127. 10.1306/071002730105

[B15] Azam F. Long R. A. (2001). Oceanography – Sea snow microcosms. *Nature* 414 495–498. 10.1038/35107174 11734832

[B16] Baelum J. Borglin S. Chakraborty R. Fortney J. L. Lamendella R. Mason O. U. (2012). Deep-sea bacteria enriched by oil and dispersant from the Deepwater Horizon spill. *Environ. Microbiol.* 14 2405–2416. 10.1111/j.1462-2920.2012.02780.x 22616650

[B17] Bao W.-Y. Satuito C. G. Yang J.-L. Kitamura H. (2007). Larval settlement and metamorphosis of the mussel *Mytilus galloprovincialis* in response to biofilms. *Mar. Biol.* 150 565–574. 10.1007/s00227-006-0383-4

[B18] Barnhart M. M. Chapman M. R. (2006). Curli biogenesis and function. *Annu. Revs. Microbiol.* 60 131–147. 10.1146/annurev.micro.60.080805.142106 16704339 PMC2838481

[B19] Battin T. J. von der Kammer F. Weilhartner A. Ottofuelling S. Hofmann T. (2009). Nanostructured TiO2: transport behavior and effects on aquatic microbial communities under environmental conditions. *Environ. Sci. Technol.* 43 8098–8104. 10.1021/es9017046 19924929

[B20] Bauer J. E. Druffel E. R. M. Wolgast D. M. Griffin S. (2002). Temporal and regional variability in sources and cycling of DOC and POC in the northwest Atlantic continental shelf and slope. *Deep Sea Res. II* 49 4387–4419. 10.1016/S0967-0645(02)00123-6

[B21] Baumgartner L. K. Reid R. P. Dupraz C. Decho A. W. Buckley D. H. Spear J. R. (2006). Sulfate reducing bacteria in microbial mats: changing paradigms, new discoveries. *Sed. Geol.* 185 131–145. 10.1016/j.sedgeo.2005.12.008

[B22] Beech I. B. Cheung C. W. S. (1995). Interactions of EPS produced by sulphate-reducing bacteria with metal ions. *Int. Biodeter. Biodegrad.* 35 59–72. 10.1016/0964-8305(95)00082-G

[B23] Bejar V. Calvo C. Moliz J. Diaz-Martinez F. Quesada E. (1996). Effect of growth conditions on the rheological properties and chemical composition of *Volcaniella eurihalina* exopolysaccharide. *Appl. Biochem. Biotechnol.* 59 77–86. 10.1007/BF02787859

[B24] Benner R. (2002). “Chemical composition and reactivity,” in *Biogeochemistry of Marine Dissolved Organic Matter* eds Hansell D. A. Carslon C. A. (San Diego, CA: Academic Press) 59–90. 10.1146/annurev-marine-010213-135126

[B25] Benner R. Amon R. M. W. (2015). The size-reactivity continuum of major bioelements in the ocean. *Annu. Rev. Mar. Sci.* 7 185–205. 10.1146/annurev-marine-010213-135126 25062478

[B26] Benner R. Pakulski J. D. McCarthy M. Hedges J. I. Hatcher P. G. (1992). Bulk chemical characteristics of dissolved organic matter in the ocean. *Science* 255 1561–1564. 10.1126/science.255.5051.1561 17820170

[B27] Benninghoff J. C. Wingender J. Flemming H.-C. Siebers B. (2016). “Biofilms X-treme: composition of extracellular polymeric substances in Archaea,” in *The Perfect Slime -Microbial Extracellular Polymeric Substances (EPS)* eds Flemming H.-C. Neu T. R. Wingender J. (London: IWA Publishers) 301–317.

[B28] Benzerara K. Menguy N. Lopez-Garcia P. Yoon T.-H. Kazmierczak J. Typiszczak T. (2006). Nanoscale detection of organic signatures in carbonate microbialites. *Proc. Natl. Acad. Sci. U.S.A.* 103 9440–9445. 10.1073/pnas.0603255103 16772379 PMC1480426

[B29] Bernhard J. M. Bowser S. S. (1992). Bacterial biofilms as a trophic resource for certain benthic foraminifera. *Mar. Ecol. Prog. Ser.* 83 263–272. 10.3354/meps083263

[B30] Bhaskar P. V. Bhosle N. B. (2005). Microbial extracellular polymeric substances in marine biogeochemical processes. *Curr. Sci.* 88 45–53.

[B31] Bhaskar P. V. Bhosle N. B. (2006). Bacterial extracellular poly- meric substances (EPS): a carrier of heavy metals in the marine food-chain. *Environ. Int.* 32 191–198. 10.1016/j.envint.2005.08.010 16256198

[B32] Bianchi M. Marty D. Teyssié J. L. Fowler S. W. (1992). Strictly aerobic and anaerobic bacteria associated with sinking particulate matter and zooplankton fecal pellets. *Mar. Ecol. Prog. Ser.* 88 55–60. 10.3354/meps088055

[B33] Biermann C. J. (1988). Hydrolysis and other cleavages of glycosidic linkages in polysaccharides. *Adv. Carbohydr. Chem. Biochem.* 46 251–271. 10.1016/S0065-2318(08)60168-7

[B34] Bigg E. K. (2007). Sources, nature, and influence on climate of marine airborne particulates. *Environ. Chem.* 4 155–161. 10.1071/EN07001

[B35] Bigg E. K. Leck C. (2008). The composition of fragments of bubbles bursting at the ocean surface. *J. Geophys. Res.* 113 D11209 10.1029/2007jd009078

[B36] Biller S. J. Schubotz F. Roggensack S. E. Thompson A. W. Summons R. E. Chisholm S. W. (2014). Bacterial vesicles in marine ecosystems. *Science* 343 183–186. 10.1126/science.1243457 24408433

[B37] Böckelmann U. Janke A. Kuhn R. Neu T. R. Wecke J. Lawrence J. R. (2005). Bacterial extracellular DNA forming a defined network-like structure. *FEMS Microbiol. Lett.* 262 31–38. 10.1111/j.1574-6968.2006.00361.x 16907736

[B38] Boehm P. D. Fiest D. L. (1980). “Aspects of the transport of petroleum hydrocarbons to the offshore benthos during the Ixtoc-I blowout in the Bay of Campeche,” in *Proceedings of the Symposium on the Preliminary Results from the September, 1979 Pierce/Research IXTOC-1Cruises* (Boulder, CO: NOAA).

[B39] Boetius A. Anesio A. M. Deming J. W. Mikucki J. A. Rapp J. Z. (2015). Microbial ecology of the cryosphere: sea ice and glacial habitats. *Nat. Revs. Microbiol.* 13 677–690. 10.1038/nrmicro3522 26344407

[B40] Bouton A. Vennin E. Boulle J. Pace A. Bourillot R. Thomazo C. (2016). Linking the distribution of microbial deposits from the Great Salt Lake (Utah, USA) to tectonic and climatic processes. *Biogeosciences* 13 5511–5526. 10.5194/bg-13-5511-2016

[B41] Boyd P. W. Jickells T. Law C. S. Blain S. Boyle E. A. Buesseler K. O. (2007). Mesoscale iron enrichment experiments 1993–2005: synthesis and future directions. *Science* 315 612–617. 10.1126/science.1131669 17272712

[B42] Braissant O. Cailleau G. Dupraz C. Verrechia E. P. (2003). Bacterially induced mineralization of calcium carbonate in terrestrial environments: the role of exopolysaccharides and amino acids. *J. Sed. Res.* 73 485–490. 10.1306/111302730485

[B43] Braissant O. Decho A. W. Dupraz C. Glunk C. Przekop K. M. Visscher P. T. (2007). Exopolymeric substances of sulfate-reducing bacteria: Interactions with calcium at alkaline pH and implication for formation of carbonate minerals. *Geobiology* 5 401–411. 10.1111/j.1472-4669.2007.00117.x

[B44] Braissant O. Decho A. W. Przekop K. M. Gallagher K. L. Glunk C. Dupraz C. (2009). Characteristics and turnover of exopolymeric substances in a hypersaline microbial mat. *FEMS Microbiol. Ecol.* 67 293–307. 10.1111/j.1574-6941.2008.00614.x 19049495

[B45] Brogioli D. Vailati A. (2001). Diffusive mass transfer by non-equilibrium fluctuations: Fick’s Law revisited. *Phys. Revs.* 63:012105 10.1103/PhysRevE.63.012105 11304296

[B46] Brussaard C. P. D. Bidle K. D. Pedrós-Alió C. Legrand C. (2016). The interactive microbial ocean. *Nat. Microbiol.* 2:16255 10.1038/nmicrobiol.2016.255 27995930

[B47] Burne R. V. Moore L. S. (1987). Microbialites: organosedimentary deposits of benthic microbial communities. *Palaios* 2 241–254. 10.2307/3514674

[B48] Camacho-Chab J. C. Lango-Reynoso F. del Refugio Castañeda-Chávez M. Galaviz-Villa I. Hinojosa-Garro D. Ortega-Morales B. O. (2016). Implications of extracellular polymeric substance matrices of microbial habitats associated with coastal aquaculture systems. *Water* 8:369 10.3390/w8090369

[B49] Camilli A. Bassler B. L. (2006). Bacterial small-molecule signaling pathways. *Science* 311 1113–1116. 10.1126/science.1121357 16497924 PMC2776824

[B50] Carlson C. A. Ducklow H. W. Michaels A. F. (1994). Annual flux of dissolved organic carbon from the euphotic zone in the northwestern Sargasso Sea. *Nature* 371 405–408. 10.1038/371405a0

[B51] Carlson C. A. Giovannoni S. J. Hansell D. A. Goldberg S. J. Parsons R. Vergin K. (2004). Interactions among dissolved organic carbon, microbial processes, and community structure in the mesopelagic zone of the northwestern Sargasso Sea. *Limnol. Oceanogr.* 49 1073–1083. 10.4319/lo.2004.49.4.1073

[B52] Caron D. A. Lim E. L. Sanders R. W. Dennett M. R. Berninger U. G. (2000). Responses of bacterioplankton and phytoplankton to organic carbon and inorganic nutrient additions in contrasting oceanic ecosystems. *Aquat. Microbiol. Ecol.* 22 175–184. 10.3354/ame022175

[B53] Chagas A. A. P. Webb G. E. Burne R. V. Southam G. (2016). Modern lacustrine microbialites: toward a synthesis of aqueous and carbonate geochemistry and mineralogy. *Earth Sci. Revs.* 162 338–363. 10.1016/j.earscirev.2016.09.012

[B54] Chew S. C. Kundukad B. Seviour T. van der Maarel J. R. C. Yang L. Rice S. A. (2014). Dynamic remodeling of microbial biofilms by functionally distinct exopolysaccharides. *MBio* 5:e1536-14 10.1128/mBio.01536-14 PMC4128364 25096883

[B55] Chin W.-C. Orellana M. V. Verdugo P. (1998). Spontaneous assembly of marine dissolved organic matter into polymer gels. *Nature* 391 568–572. 10.1038/35345

[B56] Church M. J. (2008). “Resource control of bacterial dynamics in the sea,” in *Microbial Ecology of the Oceans* 2nd Edn ed. Kirchman D. L. (Hoboken, NJ: John Wiley & Sons, Inc).

[B57] Churchill M. E. A. Chen L. (2011). Structural basis of acyl-homoserine lactone-dependent signaling. *Chem. Rev.* 111 68–85. 10.1021/cr1000817 21125993 PMC3494288

[B58] Collins R. E. Carpenter S. D. Deming J. W. (2008). Spatial heterogeneity and temporal dynamics of particles, bacteria, and pEPS in Arctic winter sea ice. *J. Mar. Syst.* 74 902–917. 10.1016/j.jmarsys.2007.09.005

[B59] Costerton J. W. Cheng K. J. Geesey G. G. Ladd T. I. Nickel J. C. Dasgupta M. (1987). Bacterial biofilms in nature and disease. *Annu. Rev. Microbiol.* 41 435–464. 10.1146/annurev.mi.41.100187.002251 3318676

[B60] Cotner J. B. Ammerman J. W. Peele E. R. Bentzen E. (1997). Phosphorus-limited bacterioplankton growth in the Sargasso Sea. *Aquat. Microbiol. Ecol.* 13 141–149. 10.3354/ame013141

[B61] Daly K. L. Passow U. Chanton J. Hollander D. (2016). Assessing the impacts of oil-associated marine snow formation and sedimentation during and after the Deepwater Horizon oil spill. *Anthropocene* 13 18–33. 10.1016/j.ancene.2016.01.006

[B62] Davies J. Spiegelman G. B. Grace Y. (2006). The world of subinhibitory antibiotic concentrations. *Curr. Opin. Microbiol.* 9 445–453. 10.1016/j.mib.2006.08.006 16942902

[B63] De Beer D. Stoodley P. Roe F. Lewandowski Z. (2004). Effects of biofilm structure on oxygen distribution and mass transport. *Biotechnol. Bioeng.* 43 1131–1133. 10.1002/bit.260431118 18615526

[B64] De Jong E. van Rens L. Westbroek P. Bosch L. (1979). Biocalcification by the marine alga *Emiliania huxleyi* (Lohmann) Kamptner. *Eur. J. Biochem.* 99 559–567. 10.1111/j.1432-1033.1979.tb13288.x 499216

[B65] de Nys R. Steinberg P. D. Willemsen P. Dworjanyn S. A. Gabelish C. L. King R. J. (2009). Broad spectrum effects of secondary metabolites from the red alga *Delisea pulchra* in antifouling assays. *Biofouling* 8 259–271. 10.1080/08927019509378279

[B66] Decho A. W. (1990). Microbial exopolymer secretions in ocean environments: their role(s) in food webs and marine processes. *Oceanogr. Mar. Biol. Ann. Rev.* 28 73–153.

[B67] Decho A. W. (1999). Imaging an alginate polymer gel matrix using atomic force microscopy. *Carbohydr. Res.* 315 330–333. 10.1016/S0008-6215(99)00006-3

[B68] Decho A. W. (2000a). “Exopolymer microdomains as a structuring agent for heterogeneity with microbial biofilms,” in *Microbial Sediments* eds Riding R. E. Awramik S. M. (Berlin: Springer-Verlag Press) 9–15.

[B69] Decho A. W. (2000b). Microbial biofilms in intertidal systems: an overview. *Cont. Shelf Res.* 20 1257–1273. 10.1016/S0278-4343(00)00022-4

[B70] Decho A. W. (2010). Overview of biopolymer-induced mineralization: what goes on in biofilms? *Ecol. Eng.* 36 137–144. 10.1016/j.ecoleng.2009.01.003

[B71] Decho A. W. (2015). “Localization of quorum sensing by extracellular polymeric substances (EPS): considerations of in situ signaling,” in *The Physical Basis of Bacterial Quorum Communication* ed. Hagen S. J. (New York, NY: Springer) 10.1007/978-1-4939-1402-9_6

[B72] Decho A. W. (2016). “Unique and baffling aspects of the matrix: EPS syneresis and glass formation during desiccation,” in *The Perfect Slime* eds Flemming H.-C. Neu T. R. Wingender J. (London: IWA Publishing) 207–226. 10.2166/9781780407418

[B73] Decho A. W. Frey R. L. Ferry J. L. (2011). Chemical challenges to bacterial AHL signaling in the environment. *Chem. Rev.* 111 86–99. 10.1021/cr100311q 21142012

[B74] Decho A. W. Kawaguchi T. Allison M. A. Louchard E. M. Reid R. P. Stephens C. (2003). Sediment properties influencing upwelling spectral reflectance signatures: the “biofilm gel effect”. *Limnol. Oceanogr.* 48 431–443. 10.4319/lo.2003.48.1_part_2.0431

[B75] Decho A. W. Lopez G. R. (1993). Exopolymer microenvironments of microbial flora: multiple and interactive effects on trophic relationships. *Limnol. Oceanogr.* 38 1633–1645. 10.4319/lo.1993.38.8.1633

[B76] Decho A. W. Moriarty D. J. W. (1990). Bacterial exopolymer utilization by a harpacticoid copepod: a methodology and results. *Limnol. Oceanogr.* 35 1039–1049. 10.4319/lo.1990.35.5.1039

[B77] Decho A. W. Norman R. S. Visscher P. T. (2010). Quorum sensing in natural environmental: emerging views from microbial mats. *Trends Microbiol.* 18 73–80. 10.1016/j.tim.2009.12.008 20060299

[B78] Decho A. W. Visscher P. T. Ferry J. Kawaguchi T. He L. Przekop K. M. (2009). Autoinducers extracted from microbial mats reveal a surprising diversity of N-acylhomoserine lactones (AHLs) and abundance changes that may relate to diel pH. *Environ. Microbiol.* 11 409–420. 10.1111/j.1462-2920.2008.01780.x 19196272

[B79] Decho A. W. Visscher P. T. Reid R. P. (2005). Production and cycling of natural microbial EPS (EPS) within a marine stromatolite. *Palaeogeogr. Palaeoclimat. Palaeoecol.* 219 71–86. 10.1016/j.palaeo.2004.10.015

[B80] Dell’Anno A. Corinaldesi C. (2004). Degradation and turnover of extracellular DNA in marine sediments: ecological and methodological considerations. *Appl. Environ. Microbiol.* 70 4384–4386. 10.1128/AEM.70.7.4384-4386.2004 15240325 PMC444808

[B81] Dell’Anno A. Danovaro R. (2005). Extracellular DNA plays a role in deep-sea ecosystem functioning. *Science* 309 2179 10.1126/science.1117475 16195451

[B82] DeLong E. F. Franks D. G. Alldredge A. L. (1993). Phylogenetic diversity of aggregate-attached vs. free-living marine bacterial assemblages. *Limnol. Oceanogr.* 38 924–934. 10.4319/lo.1993.38.5.0924

[B83] DePas W. H. Syed A. K. Sifuentes M. Lee J. S. Warshaw D. Saggar V. (2014). Biofilm formation protects *Escherichia coli* against killing by *Caenorhabditis elegans* and *Myxococcus xanthus*. *Appl. Environ. Microb.* 80 7079–7087. 10.1128/AEM.02464-14 PMC4248994 25192998

[B84] Des Marais D. J. (2003). Biogeochemistry of hypersaline microbial mats illustrates the dynamics of modern microbial ecosystems and the early evolution of the biosphere. *Biol. Bull.* 204 160–167. 10.2307/1543552 12700147

[B85] Deschatre M. Ghillebaert F. Guezennec J. Colin C. S. (2013). Sorption of copper(II) and silver(1) by four bacterial exopolysaccharides. *Appl. Biochem. Biotechnol.* 171 1313–1327. 10.1007/s12010-013-0343-7 23949682

[B86] Devries A. L. (1971). Glycoproteins as biological antifreeze agents in antarctic fishes. *Science* 172 1152–1155. 10.1126/science.172.3988.1152 5574522

[B87] Dickson J. L. Head J. W. Levy J. S. Marchant D. R. (2013). Don Juan Pond, Antarctica: near-surface CaCl2-brine feeding Earth’s most saline lake and implications for Mars. *Sci. Rep.* 3:1166 10.1038/srep01166 PMC3559074 23378901

[B88] Dohnalkova A. C. Marshall M. J. Arey B. W. Williams K. H. Buck E. C. Fredrickson J. K. (2011). Imaging hydrated microbial extracellular polymers: comparative analysis by electron microscopy. *Appl. Environ. Microbiol.* 77 1254–1262. 10.1128/AEM.02001-10 21169451 PMC3067245

[B89] Dorosz J. N. A. Castro-Mejia J. L. Hansen L. H. Nielsen D. S. Skovgaard A. (2016). Different microbiomes associated with the copepods *Acartia tonsa* and *Temora longicornis* from the same marine environment. *Aquat. Microb. Ecol.* 78 1–9. 10.3354/ame01799

[B90] Drake L. A. Doblin M. A. Dobbs F. C. (2007). Potential microbial bioinvasions via ships’ ballast water, sediment, and biofilm. *Mar. Poll. Bull.* 55 333–341. 10.1016/j.marpolbul.2006.11.007 17215010

[B91] Dupraz C. Fowler A. Tobias C. Visscher P. T. (2013). Stomatolitic knobs in Storr’s Lake (San Salvador, Bahamas): a model system for formation and alteration of laminae. *Geobiology* 11 527–548. 10.1111/gbi.12063 24118887

[B92] Dupraz C. Reid R. P. Braissant O. Decho A. W. Norman R. S. Visscher P. T. (2009). Processes of carbonate precipitation in modern microbial mats. *Earth Sci. Revs.* 96 141–162. 10.1016/j.earscirev.2008.10.005

[B93] Dupraz C. Visscher P. T. (2005). Microbial lithification in marine stromatolites and hypersaline mats. *Trends Microbiol.* 13 429–438. 10.1016/j.tim.2005.07.008 16087339

[B94] Dupraz C. Visscher P. T. Baumgartner L. K. Reid R. P. (2004). Microbe-mineral interactions: early carbonate precipitation in a hypersaline lake (Eleuthera Island, Bahamas). *Sedimentology* 51 745–765. 10.1111/j.1365-3091.2004.00649.x

[B95] Dupuy C. Mallet C. Guizien K. Montanié H. Bréret M. Mornet F. (2014). Sequential resuspension of biofilm components (Viruses, prokaryotes and protists) as measured by erodimetry experiments in the Brouage mudflat (French Atlantic coast). *J. Sea Res.* 92 56–65. 10.1016/j.seares.2013.12.002

[B96] Elhanawy W. Debelyy M. O. Feldman M. F. (2014). Preferential packing of acidic glycosidases and proteases into bacteriodes outer membrane vesicles. *mBio* 5:e00909-14 10.1128/mBio.00909-14 PMC3952158 24618254

[B97] Engel A. Passow U. (2001). Carbon and nitrogen content of transparent exopolymer particles (TEP) in relation to their Alcian Blue adsorption. *Mar. Ecol. Progr. Ser.* 219 1–10. 10.3354/meps219001

[B98] Engel A. Thoms S. Riebesell U. Rochelle-Newall E. Zondervan I. (2004). Polysaccharide aggregation as a potential sink of marine dissolved organic carbon. *Nature* 428 929–932. 10.1038/nature02453 15118723

[B99] Ewert M. Deming J. W. (2014). Bacterial responses to fluctuations and extremes in temperature and brine salinity at the surface of Arctic winter sea ice. *FEMS Microbiol. Ecol.* 89 476–489. 10.1111/1574-6941.12363 24903191

[B100] Fabbri S. Stoodley P. (2016). “Mechanical properties of biofilms,” in *The Perfect Slime* eds Flemming H.-C. Neu T. R. Wingender J. (London: IWA Publishing) 153–177. 10.2166/9781780407418

[B101] Fabrega J. Zhang R. Renshaw J. C. Liu W.-T. Lead J. R. (2011). Impact of silver nanoparticles on natural marine biofilm bacteria. *Chemosphere* 85 961–966. 10.1016/j.chemosphere.2011.06.066 21782209

[B102] Facchini M. C. Rinaldi M. Decesari S. Carbone C. Finessi E. Mircea M. (2008). Primary submicron marine aerosol dominated by insoluble organic colloids and aggregates. *Geophys. Res. Lett.* 35 L17814 10.1029/2008gl034210

[B103] Fandrich M. (2007). On the structural definition of amyloid fibrils and other polypeptide aggregates. *Cell. Mol. Life Sci.* 64 2066–2078. 10.1007/s00018-007-7110-2 17530168 PMC11138455

[B104] Fernandez A. B. Rasuk M. C. Visscher P. T. Contreras M. Novoa F. Poire D. G. (2016). Microbial diversity in sediment ecosystems (evaporate domes, microbial mats and crusts) of hypersaline Laguna Tebenquiche, Salar de Atama, Chile. *Front. Microbiol.* 7:1284 10.3389/fmicb.2016.01284 PMC4992683 27597845

[B105] Ferrer R. P. Zimmer R. K. (2012). Community ecology and the evolution of molecules of keystone significance. *Biol. Bull.* 223 167–177. 10.1086/BBLv223n2p167 23111129

[B106] Ferry J. L. Craig P. Hexel C. Sisco P. Frey R. Pennington P. L. (2009). Transfer of gold nanoparticles from the water column to the estuarine food web. *Nat. Nanotechnol.* 4 441–444. 10.1038/nnano.2009.157 19581897

[B107] Flemming H.-C. (2016). “The perfect slime – and the ‘dark matter’ of biofilms,” in *The Perfect Slime: Microbial Extracellular Polymeric Substances (EPS)* eds Flemming H.-C. Neu T. R. Wingender J. (London: IWA Publishers) 1–14. 10.2166/9781780407418

[B108] Flemming H. C. Neu T. R. Wozniak D. (2007). The EPS matrix: The house of biofilm cells. *J. Bacteriol.* 189 7945–7947. 10.1128/JB.00858-07 17675377 PMC2168682

[B109] Flemming H. C. Wingender J. (2010). The biofilm matrix. *Nat. Rev. Microbiol.* 8 623–633. 10.1038/nrmicro2415 20676145

[B110] Flemming H.-C. Wingender J. Kjelleberg S. Steinberg P. Rice S. Szewzyk U. (2016). Biofilms: an emergent form of microbial life. *Nat. Rev. Microbiol.* 14 563–575. 10.1038/nrmicro.2016.94 27510863

[B111] Fong J. N. C. Yildiz F. H. (2015). Biofilm matrix proteins. *Microbiol. Spectr*. 3 10.1128/microbiolspec.MB-0004-2014 PMC4480581 26104709

[B112] Ford T. Sacco E. Black J. Kelley T. Goodacre R. C. Berkeley R. C. W. (1991). Characterization of EPS of aquatic bacteria by pyrolysis-mass spectrometry. *Appl. Environ. Microbiol.* 57 1595–1601. 11536484 10.1128/aem.57.6.1595-1601.1991 PMC183438

[B113] Franks A. Egan S. Holmstrom C. James S. Lappin-Scott H. Kjelleberg S. (2006). Inhibition of fungal colonization by *Pseudoalteromonas tunicata* provides a competitive advantage during surface colonization. *Appl. Environ. Microbiol.* 72 6079–6087. 10.1128/AEM.00559-06 16957232 PMC1563610

[B114] Franks J. Stolz J. F. (2009). Flat laminated microbial mat communities. *Earth Sci. Rev.* 96 163–172. 10.1016/j.earscirev.2008.10.004

[B115] Frey R. L. He L. Cui Y. Decho A. W. Kawaguchi T. Ferguson P. L. (2010). Reaction of N-acylhomoserine lactones with hydroxyl radicals: rates, products, and effects on signaling activity. *Environ. Sci. Technol.* 44 7465–7469. 10.1021/es100663e 20809590

[B116] Fu J. Gong Y. Zhao X. O’Reilly S. E. Zhao D. (2014). Effects of oil and dispersants on formation of marine oil snow and transport of oil hydrocarbons. *Environ. Sci. Technol.* 48 14392–14399. 10.1021/es5042157 25420231

[B117] Fuentes E. Coe H. Green D. de Leeuw G. McFiggans G. (2010). On the impacts of phytoplankton-derived organic matter on the production of marine aerosol – Part 1: source fluxes. *Atmos. Chem. Phys.* 10 9295–9317. 10.5194/acp-10-9295-2010

[B118] Fuqua W. C. Winans S. C. Greenberg E. P. (1996). Census and consensus in bacterial ecosystems: the *LuxR/Luxl* family of quorum sensing transcriptional regulators. *Annu. Rev. Microbiol.* 50 727–751. 10.1146/annurev.micro.50.1.727 8905097

[B119] Gallagher K. L. Braissant O. Kading T. J. Dupraz C. Visscher P. T. (2013). Phosphate-related artifacts in carbonate mineralization experiments. *J. Sed. Res.* 83 37–49. 10.2110/jsr.2013.9

[B120] Gallagher K. L. Dupraz C. Visscher P. T. (2014). Two opposing effects of sulfate reduction on carbonate precipitation in normal marine, hypersaline, and alkaline environments – Comment. *Geology* 42 e313–e314. 10.1130/g34639c.1

[B121] Gallagher K. L. Kading T. J. Braissant O. Dupraz C. Visscher P. T. (2012). Inside the alkalinity engine: the role of electron donors in the organomineralization potential of sulfate-reducing bacteria. *Geobiology* 10 518–530. 10.1111/j.1472-4669.2012.00342.x 22925453

[B122] Gautret P. Camoin G. Golubic S. Sprachta S. (2004). Biochemical control of calcium carbonate precipitation in modern lagoonal microbialites, Tikehau atoll, French Polynesia. *J. Sed. Res.* 74 462–478. 10.1306/012304740462

[B123] Gautret P. Trichet J. (2005). Automicrites in modern cyanobacterial stromatolitic deposits of Rangiroa, Tuamotu Archipelago, French Polynesia: biochemical parameters underlaying their formation. *Sed. Geol.* 178 55–73. 10.1016/j.sedgeo.2005.03.012

[B124] Gebbink M. F. B. G. Claessen D. Bouma B. Dijkhuizen L. Wösten H. A. (2005). Amyloids – a functional coat for microorganisms. *Nat. Rev. Microbiol.* 3 333–341. 10.1038/nrmicro1127 15806095

[B125] Gerbersdorf S. U. Bittner R. Lubarsky H. Manz W. Paterson D. M. (2009). Microbial assemblages as ecosystem engineers of sediment stability. *J. Soils Sed.* 9 640–652. 10.1007/s11368-009-0142-5

[B126] Giese B. (2002). Long-distance electron transfer through DNA. *Annu. Rev. Biochem.* 71 51–70. 10.1146/annurev.biochem.71.083101.134037 12045090

[B127] Gloag E. S. Turnbull L. Huang A. Vallotton P. Wang H. Nolan L. M. (2013). Self-organization of bacterial biofilms is facilitated by extracellular DNA. *Proc. Natl. Acad. Sci. U.S.A.* 110 11541–11546. 10.1073/pnas.1218898110 23798445 PMC3710876

[B128] Glunk C. Dupraz C. Braissant O. Gallagher K. L. Verrecchia E. P. Visscher P. T. (2009). Microbially mediated carbonate precipitation in a hypersaline lake, Big Pond (Eleuthera, Bahamas). *Sedimentology* 58 720–736. 10.1111/j.1365-3091.2010.01180.x

[B129] Goh F. Allen M. A. Leuko S. Kawaguchi T. Decho A. W. Burns B. P. (2009). Determining the specific microbial populations and their spatial distribution within the stromatolite ecosystem of Shark Bay. *ISME J.* 3 383–396. 10.1038/ismej.2008.114 19092864

[B130] Grabowski R. C. Droppo I. G. Wharton G. (2011). Erodibility of cohesive sediment: the importance of sediment properties. *Earth Sci. Rev.* 105 101–120. 10.1016/j.earscirev.2011.01.008

[B131] Gram L. Grossart H.-P. Schlingloff A. Kiorboe T. (2002). Possible quorum sensing in marine snow bacteria: production of acylated homoserine lactones by *Roseobacter* strains isolated from marine snow. *Appl. Environ. Microbiol.* 68 4111–4116. 10.1128/AEM.68.8.4111-4116.2002 12147515 PMC123997

[B132] Grant J. Gust G. (1987). Prediction of coastal sediment stability from photopigment content of mats of purple sulfur bacteria. *Nature* 330 244–246. 10.1038/330244a0

[B133] Grossart H.-P. Engel A. Arnosti C. De La Rocha C. Murray A. Passow U. (2007). Microbial dynamics in autotrophic and heterotrophic seawater mesocosms. III. Organic matter fluxes. *Aquat. Microb. Ecol.* 49 143–156. 10.3354/ame01140

[B134] Grossart H.-P. Kiørboe T. Tang K. Ploug H. (2003). Bacterial colonization of particles: growth and interactions. *Appl. Environ. Microbiol.* 69 3500–3509. 10.1128/AEM.69.6.3500-3509.2003 12788756 PMC161515

[B135] Grossart H. P. Simon M. (2002). Bacterioplankton dynamics in the Gulf of Aqaba and the Northern Red Sea in early spring. *Mar. Ecol. Prog. Ser.* 239 263–276. 10.3354/meps239263

[B136] Guezennec J. (2002). Deep-sea hydrothermal vents: a new source of innovative bacterial exopolysaccharides of biotechnological interests. *J. Indust. Microbiol. Biotechnol.* 29 204–208. 10.1038/sj.jim.7000298 12355321

[B137] Guezennec J. Herry J. M. Kouzayha A. Bachere E. Mittelman M. Fontaine M. N. B. (2012). Exopolysaccharides from unusual marine environments inhibit early stages of biofouling. *Int. Biodeterior. Biodegrad.* 66 1–7. 10.1016/j.ibiod.2011.10.004

[B138] Guezennec J. Pignet P. Lijour Y. Gentric E. Ratiskol J. Colliec-Jouault S. (1998). Sulfation and depolymerization of a bacterial exopolysaccharide of hydrothermal origin. *Carbohydr. Polymers* 37 19–24. 10.1016/S0144-8617(98)00006-X

[B139] Guiot E. Georges P. Brun A. Fontaine-Aupart M. P. Bellon-Fontaine M. N. Briandet R. (2002). Heterogeneity of diffusion inside microbial biofilms determined by fluorescence correlation spectroscopy under two-photon excitation. *Photochem. Photobiol.* 75 570–578. 10.1562/0031-8655(2002)075<0570:HODIMB>2.0.CO;2 12081317

[B140] Guizien K. Dupuy C. Ory P. Montainié H. Hartmann H. Chatelain M. (2014). Microorganism dynamics during a rising tide: disentangling effects of resuspension and mixing with offshore waters above an intertidal mudflat. *J. Mar. Syst.* 129 178–188. 10.1016/j.jmarsys.2013.05.010

[B141] Guo L. Coleman C. H. J. Santschi P. H. (1994). The distribution of colloidal and dissolved organic carbon in the Gulf of Mexico. *Mar. Chem.* 45 105–119. 10.1016/0304-4203(94)90095-7

[B142] Gutierrez T. Berry D. Yang T. Mishamandani S. McKay L. Teske A. (2013). Role of bacterial exopolysaccharides (EPS) in the fate of the oil released during the Deepwater Horizon oil spill. *PLoS ONE* 8:e67717 10.1371/journal.pone.0067717 PMC3694863 23826336

[B143] Gutierrez T. Biller D. V. Shimmield T. Green D. H. (2012). Metal binding properties of the EPS produced by *Halomonas* sp. TG39 and its potential in enhancing trace element bioavailability to eukaryotic phytoplankton. *Biometals* 25 1185–1194. 10.1007/s10534-012-9581-3 22960806

[B144] Gutierrez T. Morris G. Green D. H. (2009). Yield and physicochemical properties of EPS from *Halomonas* sp. strain TG39 identifies a role for protein and anionic residues (sulfate and phosphate) in emulsification of *n*-hexadecane. *Biotechnol. Bioeng.* 103 207–216. 10.1002/bit.22218 19160375

[B145] Gutierrez T. Mulloy B. Bavington C. Black K. Green D. H. (2007a). Partial purification and chemical characterization of a glycoprotein (putative hydrocolloid) emulsifier produced by a marine Antarctobacter species. *Appl. Microbiol. Biotechnol.* 76 1017–1026. 10.1007/s00253-007-1091-9 17641887

[B146] Gutierrez T. Shimmield T. Haidon C. Black K. Green D. H. (2007b). Glycoprotein emulsifiers from two marine *Halomonas* species: chemical and physical characterization. *J. Appl. Microbiol.* 103 1716–1727. 10.1111/j.1365-2672.2007.03407.x 17953582

[B147] Gutierrez T. Mulloy B. Black K. Green D. H. (2008). Emulsifying and metal ion binding activity of a glycoprotein exopolymer produced by *Pseudoalteromonas* sp strain TG12. *Appl. Environ. Microbiol.* 74 4867–4876. 10.1128/AEM.00316-08 18552188 PMC2519319

[B148] Hall-Stoodley L. Costerton J. W. Stoodley P. (2004). Bacterial biofilms: from the natural environment to infectious diseases. *Nat. Rev. Microbiol.* 2 95–108. 10.1038/nrmicro821 15040259

[B149] Hanlon A. R. M. Bellinger B. Haynes K. Xiao G. Hofmann T. A. Gretz M. R. (2006). Dynamics of extracellular polymeric substance (EPS) production and loss in an estuarine, diatom-dominated, microalgal biofilm over a tidal emersion-immersion period. *Limnol. Oceanogr.* 51 79–93. 10.4319/lo.2006.51.1.0079

[B150] Hansell D. A. (2002). “DOC in the global ocean carbon cycle,” in *Biogeochemistry of Marine Dissolved Organic Matter* eds Hansell D. A. Carlson C. A. (San Diego, CA: Academic Press) 685–715. 10.1016/B978-012323841-2/50017-8

[B151] Hansell D. A. Carlson C. A. (1998). Deep-ocean gradients in the concentration of dissolved organic carbon. *Nature* 395 263–268. 10.1038/26200

[B152] Harder T. Lam C. Qian P. Y. (2002). Induction of larval settlement in the polychaete *Hydroides elegans* by marine bioWlms: an investigation of monospecifc diatom Wlms as settlement cues. *Mar. Ecol. Prog. Ser.* 229 105–112. 10.3354/meps229105

[B153] Harvey R. W. Luoma S. N. (1985). Effect of adherent bacteria and bacterial cellular polymers upon assimilation by *Macoma balthica* of sediment bound Cd, Zn, and Ag. *Mar. Ecol. Prog. Ser.* 22 281–289. 10.3354/meps022281

[B154] Hassler C. S. Alasonati E. Mancuso-Nichols C. A. Slaveykova V. I. (2011a). Exopolysaccharides produced by bacteria isolated from the pelagic Southern Ocean—role of Fe binding, chemical reactivity, and bioavailability. *Mar. Chem.* 123 88–98. 10.1016/j.marchem.2010.10.003

[B155] Hassler C. S. Schoemann V. Mancuso-Nichols C. Butler E. C. V. Boyd P. W. (2011b). Saccharides enhance iron bioavailability to Southern Ocean phytoplankton. *Proc. Natl. Acad. Sci. U.S.A.* 108 1076–1081. 10.1073/pnas.1010963108 21169217 PMC3024694

[B156] Hassler C. S. Schoemann V. (2009). Bioavailability of organically bound Fe to model phytoplankton of the Southern Ocean. *Biogeosciences* 6 2281–2296. 10.5194/bg-6-2281-2009

[B157] Hawkins L. N. Russell L. M. (2010). Polysaccharides, proteins, and phytoplankton fragments: four chemically distinct types of marine primary organic aerosol classified by single particle spectromicroscopy. *Adv. Meteorol.* 2010 612132 10.1155/2010/612132

[B158] Hedges J. I. (2002). “Why dissolved organic matter,” in *Biochemistry of Marine Dissolved Organic Matter* eds Hansell D. A. Carlson C. A. (San Diego, CA: Academic Press) 685–715.

[B159] Hense B. A. Kuttler C. Muller J. Rothballer M. Hartmann A. Kreft J.-U. (2007). Does efficiency sensing unify diffusion and quorum sensing? *Nat. Rev. Microbiol.* 5 230–239. 17304251 10.1038/nrmicro1600

[B160] Herndl G. (1988). Ecology of amorphous aggregations (marine snow) in the northern Adriatic Sea: 2. Microbial density and activity in marine snow and its implication to overall pelagic processes. *Mar. Ecol. Prog. Ser.* 48 265–275. 10.3354/meps048265

[B161] Hmelo L. Mincer T. J. Van Mooy B. A. S. (2011). Possible influence of bacterial quorum sensing on the hydrolysis of sinking particulate organic carbon in marine environments. *Environ. Microbiol. Rep.* 3 682–688. 10.1111/j.1758-2229.2011.00281.x 23761357

[B162] Hmelo L. van Mooy B. A. S. (2009). Kinetic constraints on acylated homoserine lactone-based quorum sensing in marine environments. *Aquat. Microb. Ecol.* 54 127–133. 10.3354/ame01261

[B163] Hobley L. Harkins C. MacPhee C. E. Stanley-Wall N. R. (2015). Giving structure to the biofilm matrix: and overview of individual strategies and emerging common themes. *FEMS Microbiol. Rev.* 39 649–669. 10.1093/femsre/fuv015 25907113 PMC4551309

[B164] Hobley L. Ostrowski A. Rao F. V. Bromley K. M. Porter M. Prescott A. R. (2013). BslA is a self-assembling bacterial hydrophobin that coats the *Bacillus subtilus* biofilm. *Proc. Natl. Acad. Sci. U.S.A.* 110 13600–13605. 10.1073/pnas.1306390110 23904481 PMC3746881

[B165] Holbrook R. D. Murphy K. E. Morrow J. B. Cole K. D. (2008). Trophic transfer of nanoparticles in a simplified invertebrate food web. *Nat. Nanotechnol.* 3 352–355. 10.1038/nnano.2008.110 18654546

[B166] Holmström C. Egan S. Franks A. McCloy S. Kjelleberg S. (2002). Antifouling activities expressed by marine surface associated *Pseudoalteromonas* species. *FEMS Microbiol. Ecol.* 41 47–58. 10.1111/j.1574-6941.2002.tb00965.x 19709238

[B167] Holmström C. Rittschof D. Kjelleberg S. (1992). Inhibition of settlement by larvae of *Balanus amphitrite* and *Ciona intestinalis* by a surface-colonizing marine bacterium. *Appl. Environ. Microbiol.* 58 2111–2115. 16348728 10.1128/aem.58.7.2111-2115.1992 PMC195742

[B168] Hoppe H.-G. Arnosti C. Herndl G. F. (2001). “Ecological significance of bacterial enzymes in the marine environment,” in *Enzymes in the Environment* eds Burns R. G. Dick R. P. (Basel: Marcel Dekker) 73–107.

[B169] Hoppe H.-G. Gocke K. Koppe R. Begler C. (2002). Bacterial growth and primary production along a north-south transect of the Atlantic Ocean. *Nature* 416 168–171. 10.1038/416168a 11894092

[B170] Horswill A. R. Stoodley P. Stewart P. S. Parsek M. R. (2007). The effect of the chemical, biological, and physical environment on quorum sensing in structured microbial communities. *Anal. Bioanal. Chem.* 387 371–380. 10.1007/s00216-006-0720-y 17047948 PMC1797063

[B171] Hoskins D. L. Stancyk S. E. Decho A. W. (2003). Utilization of algal and bacterial extracellular polymeric secretions (EPS) by the deposit-feeding brittlestar *Amphipholis gracillima* (Echinodermata). *Mar. Ecol. Prog. Ser.* 247 93–101. 10.3354/meps247093

[B172] Houry A. Gohar M. Deschamps J. Tischendko E. Aymerich S. Gruss A. (2012). Bacterial swimmers that infiltrate and take over the biofilm matrix. *Proc. Natl. Acad. Sci. U.S.A.* 109 13088–13093. 10.1073/pnas.1200791109 22773813 PMC3420162

[B173] Hubas C. Sachidhanandam C. Rybarcczyk H. Lubarsky H. V. Rigaux A. Moens T. (2010). Bacterivorous nematodes stimulate microbial growth and exopolymer production in marine sediment microcosms. *Mar. Ecol. Prog. Ser.* 419 85–94. 10.3354/meps08851

[B174] Hultin K. A. H. Nilsson E. D. Krejci R. Mårtensson E. M. Ehn M. Hagström Å. (2010). *In situ* laboratory sea spray production during the Marine Aerosol Production 2006 cruise on the northeastern Atlantic Ocean. *J. Geophys. Res.* 115 D06201 10.1029/2009jd012522

[B175] Ikuma K. Decho A. W. Lau B. L. T. (2015). When nanoparticles meet biofilms- interactions guiding the environmental fate and accumulation of nanoparticles. *Front. Microbiol.* 6:591 10.3389/fmicb.2015.00591 PMC4468922 26136732

[B176] Ikuma K. Madden A. S. Decho A. W. Lau B. L. T. (2014). Deposition of nanoparticles onto polysaccharide-coated surfaces: implications for nanoparticle–biofilm interactions. *Environ. Sci. Nano* 1 117–122. 10.1039/c3en00075c

[B177] Iyer A. Mody K. Bhavanath J. (2005). Biosorption of heavy metals by a marine bacterium. *Mar. Poll. Bull.* 50 340–343. 10.1016/j.marpolbul.2004.11.012 15757698

[B178] Jatt A. N. Tang K. Liu J. Zhang Z. Zhang X.-H. (2015). Quorum sensing in marine snow and its possible influence on production of extracellular hydrolytic enzymes in marine snow bacterium *Pantoea ananatis* B9. *FEMS Microbiol. Ecol.* 91 1–13. 10.1093/femsec/fiu030 25764555

[B179] Jennings L. K. Storek K. M. Ledvina H. E. Coulon C. Marmont L. S. Sadovskaya I. (2015). Pel is a cationic exopolysaccharide that cross-links extracellular DNA in the *Pseudomonas aeruginosa* biofilm matrix. *Proc. Natl. Acad. Sci. U.S.A.* 112 11353–11358. 10.1073/pnas.1503058112 26311845 PMC4568648

[B180] Jernelöv A. Lindén O. (1981). Ixtoc I: a case study of the world’s largest oil spill. *AMBIO* 10 299–306.

[B181] Jiao N. Herndl G. J. Hansell D. A. Benner R. Kattner G. Wilhelm S. W. (2010). Microbial production of recalcitrant dissolved organic matter: long-term carbon storage in the global ocean. *Nat. Rev. Microbiol.* 8 593–599. 10.1038/nrmicro2386 20601964

[B182] Johansson S. Larsson U. Boehm P. (1980). The Tsesis oil spill impact on the pelagic ecosystem. *Mar. Pollut. Bull.* 11 284–293. 10.1016/0025-326X(80)90166-6

[B183] Johnson W. M. Soule M. C. K. Kujawinski E. B. (2016). Evidence for quorum sensing and differential metabolite production by a marine bacterium in response to DMSP. *ISME J.* 10 2304–2316. 10.1038/ismej.2016.6 26882264 PMC4989321

[B184] Joyce A. Utting S. (2015). The role of exopolymers in hatcheries: an overlooked factor in hatchery hygiene and feed quality. *Aquaculture* 446 122–131. 10.1016/j.aquaculture.2015.04.037

[B185] Kaufmann G. F. Sartorio R. Lee S.-H. Rogers C. J. Meijler M. M. Moss J. A. (2005). Revisiting quorum sensing: discovery of additional chemical and biological functions for 3-oxo-N-acylhomoserine lactones. *Proc. Natl. Acad. Sci. U.S.A.* 102 309–314. 10.1073/pnas.0408639102 15623555 PMC544315

[B186] Kawaguchi T. Decho A. W. (2002a). A laboratory investigation of cyanobacterial extracellular polymeric secretions (EPS) in influencing CaCO3 polymorphism. *J. Cryst. Growth* 240 230–235. 10.1016/S0022-0248(02)00918-1

[B187] Kawaguchi T. Decho A. W. (2002b). Characterization of extracellular polymeric secretions (EPS) from modern soft marine stromatolites (Bahamas) and its inhibitory effect on CaCO3 precipitation Prep. *Biochem. Biotechnol.* 32 51–63. 10.1081/PB-120013161 11934077

[B188] Keil R. G. Kirchman D. L. (1999). Utilization of dissolved protein and amino acids in the northern Sargasso Sea. *Aquat. Microb. Ecol.* 18 293–300. 10.3354/ame018293

[B189] Kennedy A. F. D. Sutherland I. W. (1987). Analysis of bacterial exopolysaccharides. *Biotechnol. Appl. Biochem.* 9 12–19. 3032214

[B190] Kirchman D. L. Meon B. Ducklow H. W. Carlson C. A. Hansell D. A. Steward G. F. (2001). Glucose fluxes and concentrations of dissolved combined neutral sugars (polysaccharides) in the Ross Sea and Polar Front Zone, Antarctica. *Deep Sea Res. II* 48 4179–4197. 10.1016/S0967-0645(01)00085-6

[B191] Kleindienst S. Seidel M. Ziervogel K. Grim S. Loftis K. Harrison S. (2015). Chemical dispersants can suppress the activity of natural oil-degrading microorganisms. *Proc. Natl. Acad. Sci. U.S.A.* 112 14900–14905. 10.1073/pnas.1507380112 26553985 PMC4672791

[B192] Krembs C. Eicken H. Deming J. W. (2011). Exopolymer alteration of physical properties of sea ice and implications for ice habitability and biogeochemistry in a warmer Arctic. *Proc. Natl. Acad. Sci. U.S.A.* 108 3653–3658. 10.1073/pnas.1100701108 21368216 PMC3048104

[B193] Krembs C. Eicken H. Junge K. Deming J. W. (2002). High concentrations of exopolymeric substances in Arctic winter sea ice: implication for the polar ocean carbon cycle and cryoprotection of diatoms. *Deep Sea Res. II* 49 2163–2181. 10.1016/S0967-0637(02)00122-X

[B194] Krumbein W. E. (1983). Stromatolites – the challenge of a term in space and time. *Precambr. Res.* 20 493–531. 10.1016/0301-9268(83)90087-6

[B195] Kuznetsova M. Lee C. Aller J. (2005). Characterization of the proteinaceous matter in marine aerosols. *Mar. Chem.* 96 359–377. 10.1007/s00216-012-6363-2

[B196] Lam C. Harder T. Qian P. Y. (2003). Induction of larval settlement in the polychaete *Hydroides elegans* by surface-associated settlement cues of marine benthic diatoms. *Mar. Ecol. Prog. Ser.* 263 83–92. 10.3354/meps263083

[B197] Larsen P. Nielsen J. L. Dueholm M. Wetzel R. Otzen D. Nielsen P. H. (2007). Amyloid adhesins are abundant in natural biofilms. *Environ. Microbiol.* 9 3077–3090. 10.1111/j.1462-2920.2007.01418.x 17991035

[B198] Lau S. C. K. Qian P. Y. (2000). Inhibitory effect of phenolic compounds and marine bacteria on larval settlement of the barnacle *Balanus amphitrite* Darwin. *Biofouling* 16 47–58. 10.1080/08927010009378429

[B199] Lawrence J. R. Swerhone G. D. W. Kuhlicke U. Neu T. R. (2007). In situ-evidence for microdomains in the polymer matrix of bacterial microcolonies. *Can. J. Microbiol.* 53 450–458. 10.1139/W06-146 17538657

[B200] Lawrence J. R. Swerhone G. D. W. Kuhlicke U. Neu T. R. (2016). In situ evidence for metabolic and chemical microdomains in the structured polymer matrix of bacterial microcolonies. *FEMS Microbiol. Ecol.* 92:fiw183 10.1093/femsec/fiw183 27562775

[B201] Lawrence J. R. Swerhone G. D. W. Leppard G. G. Araki T. Zhang X. West M. M. (2003). Scanning transmission X-ray, laser scanning and transmission electron microscopy mapping of the exopolymeric matrix of microbial biofilms. *Appl. Environ. Microbiol.* 69 5543–5554. 10.1128/AEM.69.9.5543-5554.2003 12957944 PMC194976

[B202] Lawrence J. R. Wolfaardt G. M. Korber D. R. (1994). Determination of diffusion coefficients in biofilms by confocal laser microscopy. *Appl. Environ. Microbiol.* 60 1166–1173. 16349228 10.1128/aem.60.4.1166-1173.1994 PMC201454

[B203] Leck C. Bigg E. K. (2005). Evolution of the marine aerosol – a new perspective. *Geophys. Res. Lett.* 32 L19803 10.1029/2005GL023651

[B204] Leck C. Bigg E. K. (2008). Comparison of sources and nature of the tropical aerosol with the summer high Arctic aerosol. *Tellus* 60B 118–126. 10.1111/j.1600-0889.2007.00315.x

[B205] Lewin R. (1984). Microbial adhesion is a sticky problem. *Science* 224 375–377. 10.1126/science.6143401 6143401

[B206] Ley R. E. Harris J. K. Wilcox J. Spear J. R. Miller S. R. Bebout B. M. (2006). Unexpected diversity and complexity from the Guerrero Negro hypersaline microbial mat. *Appl. Environ. Microbiol.* 72 3685–3695. 10.1128/AEM.72.5.3685-3695.2006 16672518 PMC1472358

[B207] Liu S.-B. Chen X.-L. He H.-L. Zhang X.-Y. Xie B.-B. Yu Y. (2013). Structure and ecological roles of a novel exopolysaccharide from the Arctic sea ice bacterium *Pseudoalteromonas* sp. Strain SM20310. *Appl. Environ. Microbiol.* 79 224–230. 10.1128/AEM.01801-12 23087043 PMC3536116

[B208] Loaec M. Olier R. Guezennec J. (1997). Uptake of lead, cadmium and zinc by a novel bacterial exopolysaccharide. *Water Res.* 31 1171–1179. 10.1016/S0043-1354(96)00375-2

[B209] Loaec M. Olier R. Guezennec J. (1998). Chelating properties of bacterial exopolysaccharides from deep-sea hydrothermal vents. *Carbohydr. Polymers* 35 65–70. 10.1016/S0144-8617(97)00109-4

[B210] Long R. A. Azam F. (1996). Abundant protein-containing particles in the sea. *Aquat. Microb. Ecol.* 10 213–221. 10.3354/ame010213

[B211] Long R. A. Azam F. (2001a). Antagonistic interactions among marine pelagic bacteria. *Appl. Environ. Microbiol.* 67 4975–4983. 11679315 10.1128/AEM.67.11.4975-4983.2001 PMC93260

[B212] Long R. A. Azam F. (2001b). Microscale patchiness of bacterioplankton assemblage richness in seawater. *Aquat. Microb. Ecol.* 26 103–113. 10.3354/ame026103

[B213] Ludwig R. Al-Horani F. A. de Beer D. Jonkers H. M. (2005). Photosynthesis controlled calcification in a hypersaline microbial mat. *Limnol. Oceanogr.* 50 1836–1843. 10.4319/lo.2005.50.6.1836

[B214] Lundqvist A. Bertilsson S. Goedkoop W. (2010). Effects of extracellular polymeric and humic substances on chlorpyrifos bioavailability to *Chironomus riparius*. *Ecotoxicology* 19 614–622. 10.1007/s10646-009-0430-2 19851864

[B215] Lundqvist A. Bertilsson S. Goedkoop W. (2012). Interactions with DOM and biofilms affect the fate and bioavailability of insecticides to invertebrate grazers. *Ecotoxicology* 21 2398–2408. 10.1007/s10646-012-0995-z 22955550

[B216] Luoma S. N. Khan F. R. Croteau M.-N. (2014). Bioavailability and bioaccumulation of metal-based engineered nanomaterials in aquatic environments: concepts and processes. *Nanosci. Environ.* 7 157–193. 10.1016/b978-0-08-099408-6.00005-0

[B217] Malarkey J. Baas J. H. Hope J. A. Aspden R. J. Parsons D. R. Peakall J. (2015). The pervasive role of biological cohesion in bedform development. *Nat. Commun.* 6:6257 10.1038/ncomms7257 PMC4347294 25656496

[B218] Martin J. H. Coale K. H. Johnson K. S. Fitzwater S. E. Gordon R. M. Tanner S. J. (1994). Testing the iron hypothesis in ecosystems of the equatorial Pacific-Ocean. *Nature* 371 123–129. 10.1038/371123a0

[B219] Marx J. G. Carpenter S. D. Deming J. W. (2009). Production of cryoprotectant extracellular polymeric substances (EPS) by the marine psychrophilic bacterium *Colwellia psychrerythraea* strain 34H under extreme conditions. *Can. J. Microbiol.* 55 63–72. 10.1139/W08-130 19190702

[B220] Mashburn L. M. Whiteley M. (2005). Membrane vesicles traffic signals and facilitate group activities in a prokaryote. *Nature* 437 422–425. 10.1038/nature03925 16163359

[B221] Mashburn-Warren L. McLean R. J. C. Whiteley M. (2008). Gram-negative outer membrane vesicles: beyond the cell surface. *Geobiology* 6 214–219. 10.1111/j.1472-4669.2008.00157.x 18459967 PMC3113409

[B222] Mayer C. Moritz R. Kirschner C. Borchard W. Maibaum R. Wingender J. (1999). The role of intermolecular interaction: studies on model systems for bacterial biofilms. *Int. J. Biol. Macromol.* 26 3–16. 10.1016/S0141-8130(99)00057-4 10520951

[B223] McLean R. J. C. Whiteley M. Stickler D. J. Fuqua W. C. (1997). Evidence of autoinducer activity in naturally occurring biofilms. *FEMS Microbiol. Lett.* 154 259–263. 10.1111/j.1574-6968.1997.tb12653.x 9311122

[B224] Meiners K. Gradinger R. Fehling J. Civitarese G. Spindler M. (2003). Vertical distribution of exopolymer particles in sea ice of the Fram Strait (Arctic) during autumn. *Mar. Ecol. Prog. Ser.* 248 1–13. 10.3354/meps248001

[B225] Meister P. (2013). Two opposing effects of sulfate reduction on carbonate precipitation in normal marine, hypersaline, and alkaline environments. *Geology* 41 499–502. 10.1130/G34185.1

[B226] Miller L. A. Papkyriakow T. N. Collins R. E. Deming J. W. Ehn J. K. MacDonald R. W. (2011). Carbon dynamics in sea ice: a winter flux time series. *J. Geophys. Res.* 116:C02028 10.1029/2009jc006058

[B227] Miller S. D. Haddock S. H. D. Elvidge C. D. Lee T. F. (2005). Detection of a bioluminescent milky sea from space. *Proc. Natl. Acad. Sci. U.S.A.* 102 14181–14184. 10.1073/pnas.0507253102 16186481 PMC1242338

[B228] Mobberley J. M. Khodadad C. L. M. Visscher P. T. Reid R. P. Hagan P. Foster J. S. (2015). Inner workings of thrombolites: spatial gradients of metabolic activity as revealed by metatranscriptome profiling. *Sci. Rep.* 5:12601 10.1038/srep12601 PMC4515876 26213359

[B229] Mopper K. Schultz C. A. Chevolot L. Germain C. Revuelta R. Dawson R. (1992). Determination of sugars in unconcentrated seawater and other natural waters by liquid chromatography and pulsed amperometric detection. *Environ. Sci. Technol.* 26 133–138. 10.1021/es00025a014

[B230] Moppert X. Le Costaouec T. Raguénès G. Simon-Colin C. Crassous P. Costa B. (2009). Investigations into the uptake of copper, iron and selenium by a highly-sulphated bacterial exopolysaccharide isolated from microbial mats. *J. Indust. Microbiol. Biotechnol.* 36 599–604. 10.1007/s10295-009-0529-8 19198908

[B231] Moran M. A. (2015). Microbiome: the global ocean microbiome. *Science* 350:aac8455 10.1126/science.aac8455 26659059

[B232] Moran M. A. Zepp R. G. (2000). “UV radiation effects on microbes and microbial processes,” in *Microbial Ecology of the Oceans* 1st Edn ed. Kirchman D. L. (Hoboken, NJ: Wile-Liss) 201–228.

[B233] Mortimer M. Petersen E. J. Buchholz B. A. Orias E. Holden P. A. (2016). Bioaccumulation of multiwall Carbon nanotubes in *Tetrahymena thermophila* by direct feeding or trophic transfer. *Environ. Sci. Technol.* 50 8876–8885. 10.1021/acs.est.6b01916 27398725 PMC4991038

[B234] Myklestad S. (1977). Production of carbohydrates by marine planktonic diatoms. II. Influence of the N/P ratio in the growth medium on the assimilation ratio, growth rate and production of cellular and extracellular carbohydrates by *Chaetoceros affinis* var Willei (Gran) Hustedt and *Skeletonema costatum* (Grev) Cleve. *J. Exp. Mar. Biol. Ecol.* 29 161–179. 10.1016/0022-0981(77)90046-6

[B235] Nagata T. (2000). “Production mechanisms of dissolved organic matter,” in *Microbial Ecology of the Oceans* 1st Edn ed. Kirchman D. L. (Hoboken, NJ: Wiley-Liss) 121–152.

[B236] Nealson K. H. Hastings J. W. (2004). Quorum sensing on a global scale: massive numbers of bioluminsescent bacteria make milky seas. *Appl. Environ. Microbiol.* 72 2295–2297. 10.1128/AEM.72.4.2295-2297.2006 PMC1448986 16597922

[B237] Neu T. R. (1994). “Biofilms and microbial mats,” in *Biostabilization of Sediments* eds Krumbein W. E. Paterson D. M. Stal L. J. (Oldenburg: BIS-Verlag) 9–17.

[B238] Neu T. R. Lawrence J. R. (2014). Advanced techniques for in situ analysis of the biofilm matrix (structure, composition, dynamics) by means of laser scanning microscopy. *Methods Mol. Biol.* 1147 43–64. 10.1007/978-1-4939-0467-9_4 24664825

[B239] Neu T. R. Lawrence J. R. (2016). “The extracellular matrix – an intractable part of biofilm systems,” in *The Perfect Slime -Microbial Extracellular Polymeric Substances (EPS)* eds Flemming H.-C. Neu T. R. Wingender J. (London: IWA Publishers) 25–60.

[B240] Nevius B. A. Chen Y. P. Chen Ferry J. L. Decho A. W. (2012). Surface-functionalization effects on uptake of fluorescent polystyrene nanoparticles by model biofilms. *Ecotoxicology* 21 2205–2213. 10.1007/s10646-012-0975-3 22806556

[B241] Nichols C. A. Guezennec J. Bowman J. P. (2005). Bacterial exopolysaccharides from extreme marine environments with special consideration of the Southern Ocean, sea ice, and deep-sea hydrothermal vents: a review. *Mar. Biotechnol.* 7 253–271. 10.1007/s10126-004-5118-2 16075348

[B242] Nichols C. M. Lardière S. G. Bowman J. P. Nichols P. D. Gibson J. A. E. Guézennec J. (2005). Chemical characterization of exopolysaccharide for Antarctic marine bacteria. *Microb. Ecol.* 49 578–589. 10.1007/s00248-004-0093-8 16052372

[B243] Nielsen S. J. Harder T. Steinberg P. D. (2015). Sea urchin larvae decipher the epiphytic bacterial community composition when selecting sites for attachment and metamorphosis. *FEMS Microbiol. Ecol.* 91 1–9. 10.1093/femsec/fiu011 25764535

[B244] Nishimura S. Tanaka T. Fujita K. Itaya M. Hiraishi A. Kikuchi Y. (2003). Extracellular DNA and RNA produced by a marine photosynthetic bacterium *Rhodovulum sulfidophilum*. *Nucleic Acids Res.* 3(Suppl.) 279–280. 10.1093/nass/3.1.279 14510489

[B245] Niu H. Li Z. Lee K. Kepkay P. Mullin J. V. (2011). Modelling the transport of oil-mineral-aggregates (OMAs) in the marine environment and assessment of their potential risks. *Environ. Model Assess.* 16 61–75. 10.1007/s10666-010-9228-0

[B246] Noffke N. (2010). *Microbial Mats in Sandy Deposits from the Archean to Today.* Heidelberg: Springer 196.

[B247] Noffke N. Christian D. Wacey D. Hazen R. M. (2013). Microbially induced sedimentary structures recording an ancient ecosystem in the *ca*. 3.48 billion-year-old dresser formation, Pilbara, Western Australia. *Astrobiology* 13 1103–1124. 10.1089/ast.2013.1030 24205812 PMC3870916

[B248] Noffke N. Gerdes G. Klenke T. Krumbein W. E. (2001). Microbially induced sedimentary structures- A new category within the classification of primary sedimentary structures. *J. Sed. Res.* 71 649–656. 10.1306/2DC4095D-0E47-11D7-8643000102C1865D

[B249] Nutman A. P. Bennett V. C. Friend C. R. L. van Kranendonk M. J. Chivas A. R. (2016). Rapid emergence of life shown by discovery of 3,700-million-year-old microbial structures. *Nature* 537 535–538. 10.1038/nature19355 27580034

[B250] Nyholm S. V. Stabb E. V. Ruby E. G. McFall-Ngai M. J. (2000). Establishment of an animal bacterial association: recruiting symbiotic vibrios from the environment. *Proc. Natl. Acad. Sci. U.S.A.* 97 10231–10235. 10.1073/pnas.97.18.10231 10963683 PMC27829

[B251] Obst M. Dynes J. J. Lawrence J. R. Swerhone G. D. W. Benzerara K. Karunakaran C. (2009). Precipitation of amorphous CaCO3 (aragonite-like) by cyanobacteria: a STXM study of the influence of EPS on the nucleation process. *Geochim. Cosmchim. Acta* 73 4180–4198. 10.1016/j.gca.2009.04.013

[B252] O’Dowd C. D. Facchini M. C. Cavalli F. Ceburnis D. Mircea M. Decesari S. (2004). Biogenically-driven organic contribution to marine aerosol. *Nature* 431 676–680. 10.1038/nature02959 15470425

[B253] Ogawa H. Amagai Y. Koike I. Kaiser K. Benner R. (2001). Production of refractory dissolved organic matter by bacteria. *Science* 292 917–920. 10.1126/science.1057627 11340202

[B254] Okshevsky M. Meyer R. L. (2013). The role of extracellular DNA in the establishment, maintenance and perpetuation of bacterial biofilms. *Crit. Revs. Microbiol.* 41 341–352. 10.3109/1040841X.2013.841639 24303798

[B255] Oren A. (1994). The ecology of extremely halophilic archaea. *FEMS Microbiol. Rev.* 13 415–439. 10.1111/j.1574-6976.1994.tb00060.x 19709178

[B256] Paerl H. W. Steppe T. F. Reid R. P. (2001). Bacterially-mediated precipitation in marine Stromatolites. *Environ. Microbiol.* 3 123–130. 10.1046/j.1462-2920.2001.00168.x 11321542

[B257] Papenfort K. Bassler B. L. (2016). Quorum sensing signal-response systems in Gram-negative bacteria. *Nat. Revs. Microbiol.* 14 576–588. 10.1038/nrmicro.2016.89 27510864 PMC5056591

[B258] Passow U. (2002). Transparent exopolymer particles (TEP) in aquatic environments. *Progr. Oceanogr.* 55 287–333. 10.1016/S0079-6611(02)00138-6

[B259] Passow U. (2016). Formation of rapidly-sinking, oil-associated marine snow. *Deep Sea Res. II Top. Stud. Oceanogr.* 129 232–240. 10.1016/j.dsr2.2014.10.001

[B260] Passow U. Ziervogel K. Asper V. Diercks A. (2012). Marine snow formation in the aftermath of the deepwater horizon oil spill in the Gulf of Mexico. *Environ. Res. Lett.* 7 1–11. 10.1088/1748-9326/7/3/035301

[B261] Paterson D. M. (1989). Short-term changes in the erodibility of intertidal cohesive sediments related to the migratory behavior of epipelic diatoms. *Limnol. Oceanogr.* 34 223–234. 10.4319/lo.1989.34.1.0223

[B262] Paterson D. M. Aspden R. J. Visscher P. T. Consalvey M. Andres M. S. Decho A. W. (2008). Light dependent biostabilisation of sediments by stromatolite assemblages. *PLoS ONE* 3:e3176 10.1371/journal.pone.0003176 PMC2526175 18781202

[B263] Patton J. S. Rigler M. W. Boehm P. D. Fiest D. L. (1981). Ixtoc I oil spill: flaking of surface mousse in the Gulf of Mexico. *Nature* 290 235–238. 10.1038/290235a0

[B264] Paul V. Mormile M. R. Wronkiewicz D. J. (2017). Impact of elevated CO2 concentrations on carbonate mineral precipitation ability of sulfate-reducing bacteria and implications for CO2 sequestration. *Appl. Geochem.* 78 250–271. 10.1016/j.apgeochem.2017.01.010

[B265] Peterson B. W. He Y. Ren Y. Zerdoum A. Libera M. R. Sharma P. K. (2015). Viscoelasticity of biofilms and their recalcitrance to mechanical and chemical challenges. *FEMS Microbiol. Rev.* 39 234–245. 10.1093/femsre/fuu008 25725015 PMC4398279

[B266] Pinckney J. L. Paerl H. W. (1997). Anoxygenic photosynthesis and nitrogen fixation by a microbial mat community in a *Bahamian* hypersaline lagoon. *Appl. Environ. Microbiol.* 63 420–426. 16535506 10.1128/aem.63.2.420-426.1997 PMC1389512

[B267] Plante C. (2000). Role of bacterial exopolymeric capsules in protection from deposit feeder ingestion. *Aquat. Microb. Ecol.* 21 211–219. 10.3354/ame021211

[B268] Plante C. J. Jumars P. A. Baross J. A. (1990). Digestive associations between marine detritivores and bacteria. *Ann. Rev. Ecol. Syst.* 21 29–44. 10.1146/annurev.es.21.110190.000521

[B269] Plante C. J. Schriver A. G. (1998). Differential lysis of sedimentary bacteria by *Arenicola marina* L.: examination of cell wall structure and exopolymeric capsules as correlates. *J. Exp. Mar. Biol. Ecol.* 229 35–52. 10.1016/S0022-0981(98)00039-2

[B270] Ploug H. (2008). Cyanobacterial surface blooms formed by *Aphanizomenon* sp. and *Nodularia spumigena* in the Baltic Sea: small-scale fluxes, pH, and oxygen micro-environments. *Limnol. Oceanogr.* 53 914–921. 10.4319/lo.2008.53.3.0914

[B271] Ploug H. Grossart H. P. Azam F. Jørgensen B. B. (1999). Photosynthesis, respiration, and carbon turnover in sinking marine snow from surface waters of the Southern California Bight: implications for the carbon cycle in the ocean. *Mar. Ecol. Prog. Ser.* 179 1–11. 10.3354/meps179001

[B272] Ploug H. Kühl M. Buchholz-Cleven B. Jørgensen B. B. (1997). Anoxic aggregates - an ephemeral phenomenon in the pelagic environment? *Aquat. Microb. Ecol.* 13 285–294. 10.3354/ame013285

[B273] Potts M. (1994). Desiccation tolerance or prokaryotes. *Microbiol. Rev.* 58 755–805. 7854254 10.1128/mr.58.4.755-805.1994 PMC372989

[B274] Raguénès G. Christen R. Guézennec J. Pignet P. Barbier G. (1997a). *Vibrio diabolicus* sp.nov., a new polysaccharide-secreting organism isolated from a deep-sea vent polychaete annelid, Alvinella pompejana. *Int. J. Syst. Bacteriol.* 47 989–995. 9336897 10.1099/00207713-47-4-989

[B275] Raguénès G. Ruimy R. Pignet P. Christen R. Loaec M. Rougeaux H. (1997b). *Alteromonas* infernus sp. nov., a new polysaccharide producing bacterium isolated from a deep-sea hydrothermal vent. *J. Appl. Bacteriol.* 82 422–430. 10.1046/j.1365-2672.1997.00125.x 9134716

[B276] Rao D. Webb J. S. Holmstrom C. Case R. Low A. Steinberg P. (2007). Low densities of epiphytic bacteria from the marine alga Ulva australis inhibit settlement of fouling organisms. *Appl. Environ. Microbiol.* 73 7844–7852. 10.1128/AEM.01543-07 17965210 PMC2168146

[B277] Redfield R. J. (2002). Is quorum sensing a side effect of diffusion sensing? *Trends Microbiol.* 10 365–370. 12160634 10.1016/s0966-842x(02)02400-9

[B278] Reid R. P. Visscher P. T. Decho A. W. Stolz J. F. Bebout B. M. Dupraz C. (2000). The role of microbes in accretion, lamination and early lithication of modern marine stromatolites. *Nature* 406 989–992. 10.1038/35023158 10984051

[B279] Repeta D. J. Aluwihare L. I. (2006). Radiocarbon analysis of neutral sugars in high-molecular-weight dissolved organic carbon: implications for organic carbon cycling. *Limnol. Oceanogr.* 51 1045–1053. 10.4319/lo.2006.51.2.1045

[B280] Rice K. C. Mann E. E. Endres J. L. Weiss E. C. Cassat J. E. Smeltzer M. S. (2007). The *cidA* murein hydrolase regulator contributes to DNA release and biofilm development in *Staphylococcus aureus*. *Proc. Natl. Acad. Sci., U.S.A.* 104 8113–8118. 10.1073/pnas.0610226104 17452642 PMC1876580

[B281] Rich J. Gosselin M. Sherr E. Sherr B. Kirchman D. L. (1997). High bacterial production, uptake and concentrations of dissolved organic matter in the Central Arctic Ocean. *Deep-Sea Res. II* 44 1645–1663. 10.1016/S0967-0645(97)00058-1

[B282] Rich J. H. Ducklow H. W. Kirchman D. L. (1996). Concentrations and uptake of neutral monosaccharides along 140°W in the Equatorial Pacific: contribution of glucose to heterotrophic bacterial activity and the POM flux. *Limnol. Oceanogr.* 41 595–604. 10.4319/lo.1996.41.4.0595

[B283] Richert L. Golubic S. Le Guédès R. Ratiskol J. Payri C. Guezennec J. (2005). Characterization of exopolysaccharides produced by cyanobacteria isolated from polynesian microbial mats. *Curr. Microbiol.* 51 379–384. 10.1007/s00284-005-0069-z 16252130

[B284] Rieger J. Frechen T. Cox G. Heckmann W. Schmidt C. Thieme J. (2007). Precursor structures in the crystallization/precipitation processes of CaCO3 and control of particle formation by polyelectrolytes. *Faraday Discuss.* 136 265–277. 10.1039/b701450c 17955814

[B285] Rivkin R. B. Anderson M. R. (1997). Inorganic nutrient limitation of oceanic bacterioplankton. *Limnol. Oceanogr.* 42 730–740. 10.1038/nature07236

[B286] Romankevich E. A. (1984). *Geochemistry of Organic Matter in the Ocean.* Berlin: Springer-Verlag 10.1007/978-3-642-49964-7

[B287] Romero D. Aguilar C. Losick R. Kolter R. (2010). Amyloid fibers provide structural integrity to *Bacillus subtilus* biofilms. *Proc. Natl. Acad. Sci. U.S.A.* 107 2230–2234. 10.1073/pnas.0910560107 20080671 PMC2836674

[B288] Rougeaux H. Guezennec M. Mao Che L. Payri C. Deslandes E. Guezennec J. (2001). Microbial communities and exopolysaccharides from Polynesian mats. *Mar. Biotechnol.* 3 181–187. 10.1007/s101260000063 14961381

[B289] Rougeaux H. Kervarec N. Pichon R. Guezennec J. (1999). Structure of the exopolysaccharide of *Vibrio diabolicus* isolated from a deep-sea hydrothermal vent. *Carbohydr. Res.* 322 40–45. 10.1016/S0008-6215(99)00214-1 10629947

[B290] Ruby E. G. Nealson K. H. (1977). Luminous bacterium that emits yellow light. *Science* 196 432–434. 10.1126/science.850787 850787

[B291] Ruparell A. Dubern J. F. Ortori C. A. Harrison F. Halliday N. M. Emtage A. (2016). The fitness burden imposed by synthesizing quorum sensing signals. *Sci. Rep.* 6:33101 10.1038/srep33101 PMC5018880 27616328

[B292] Russell L. M. Hawkins L. N. Frossard A. A. Quinn P. K. Bates T. S. (2010). Carbohydrate-like composition of submicron atmospheric particles and their production from ocean bubble bursting. *Proc. Natl. Acad. Sci. U.S.A.* 107 6652–6657. 10.1073/pnas.0908905107 20080571 PMC2872374

[B293] Salek K. Gutierrez T. (2016). Surface-active biopolymers from marine bacteria for potential biotechnological applications. *AIMS Microbiol.* 2 92–107. 10.3934/microbiol.2016.2.92

[B294] Santschi P. H. Guo L. Means J. C. Ravichandran M. (1998). “Natural organic matter binding of trace metal and trace organic contaminants in estuaries,” in *Biogeochemistry of Gulf of Mexico Estuaries* eds Bianchi T. S. Pennock J. R. Twilley R. (New York, NY: Wiley) 347–380.

[B295] Schaefer A. L. Greenberg E. P. Oliver C. M. Oda Y. Huang J. J. Bittan-Banin G. (2008). A new class of homoserine lactone quorum sensing signals. *Nature* 454 595–596. 10.1038/nature07088 18563084

[B296] Schertzer J. W. Boulette M. L. Whiteley M. (2009). More than a signal: non-signaling properties of quorum sensingmolecules. *Trends Microbiol.* 17 189–195. 10.1016/j.tim.2009.02.001 19375323

[B297] Schlekat C. E. Decho A. W. Chandler G. T. (1998). Sorption of cadmium to bacterial extracellular polymeric sediment coatings under estuarine conditions. *Environ. Toxicol. Chem.* 17 1867–1874. 10.1002/etc.5620170930

[B298] Schlekat C. E. Decho A. W. Chandler G. T. (1999). Dietary assimilation of cadmium associated with bacterial exopolymer sediment coatings by the estuarine amphipod *Leptocheirus pluinulosus*: effects of Cd concentration and salinity. *Mar. Ecol. Prog. Ser.* 183 205–216. 10.3354/meps183205

[B299] Schlekat C. E. Decho A. W. Chandler G. T. (2000). Bioavailability of particle-associated Ag, Cd, and Zn to the estuarine amphipod, Leptocheirus plumulosus, through dietary ingestion. *Limnol. Oceanogr.* 45 11–21. 10.1371/journal.pone.0064060

[B300] Schooling S. R. Hubley A. Beveridge T. J. (2009). Interaction of DNA with biofilm-derived membrane vesicles. *J. Bacteriol.* 191 4097–4102. 10.1128/JB.00717-08 19429627 PMC2698485

[B301] Selck H. Decho A. W. Forbes V. E. (1999). Effects of chronic metal exposure and sediment organic matter on digestive absorption efficiencies of cadmium by the deposit-feeding polychaete *Capitella* species I. *Environ. Toxicol. Chem.* 18 1289–1297. 10.1002/etc.5620180631

[B302] Seper A. Fengler V. H. I. Roier S. Wolinski H. Kohlwein S. D. Bishop A. L. (2011). Extracellular nucleases and extracellular DNA play important roles in *Vibrio cholera* biofilm formation. *Mol. Microbiol.* 82 1015–1037. 10.1111/j.1365-2958.2011.07867.x 22032623 PMC3212620

[B303] Serra D. O. Richter A. M. Hengge R. (2013). Cellulose as an architectural element in spatially structured *Escherichia coli* biofilms. *J. Bact.* 195 5540–5554. 10.1128/JB.00946-13 24097954 PMC3889604

[B304] Shanks A. L. Reeder M. L. (1993). Reducing microzones and sulfide production in marine snow. *Mar. Ecol. Prog. Ser.* 96 43–47. 10.3354/meps096043

[B305] Shanks A. L. Trent J. D. (1980). Marine snow: sinking rates and potential role in vertical flux. *Deep Sea Res. Part A. Oceanogr. Res. Papers* 27 137–143. 10.1016/0198-0149(80)90092-8

[B306] Sharif D. I. Gallon J. Smith C. J. Dudley E. (2008). Quorum sensing in cyanobacteria: *N*-octanoyl-homoserine lactone release and response, by the epilithic colonial cyanobacterium *Gloeotheca* PCC6909. *ISME J.* 2 1171–1182. 10.1038/ismej.2008.68 18633449

[B307] Shaw E. Hill D. R. Brittain N. Wright D. J. Täuber U. Marand H. (2003). Unusual water flux in the extracellular polysaccharide of the cyanobacterium *Nostoc commune*. *Appl Environ. Microbiol.* 69 5679–5684. 10.1128/AEM.69.9.5679 12957961 PMC194923

[B308] Simon M. Grossart H.-P. Schweitzer B. Ploug H. (2002). Microbial ecology of organic aggregates in aquatic ecosystems. *Aquat. Microb. Ecol.* 28 175–211. 10.3354/ame028175

[B309] Skoog A. Benner R. (1997). Aldoses in various size fractions of marine organic matter: implications for carbon cycling. *Limnol. Oceanogr.* 42 1803–1813. 10.4319/lo.1997.42.8.1803

[B310] Skoog A. Biddanda B. Benner R. (1999). Bacterial utilization of dissolved glucose in the upper water column of the Gulf of Mexico. *Limnol. Oceanogr.* 44 1625–1633. 10.3389/fmicb.2013.00318

[B311] Skoog A. Whitehead K. Sperling F. Junge K. (2002). Microbial glucose uptake and growth along a horizontal nutrient gradient in the North Pacific. *Limnol. Oceanogr.* 47 1676–1683. 10.4319/lo.2002.47.6.1676

[B312] Smith D. C. Simon M. Alldredge A. L. Azam F. (1992). Intense hydrolytic enzyme activity on marine aggregates and implications for rapid particle dissolution. *Nature* 359 139–142. 10.1038/359139a0

[B313] Smith D. J. Underwood G. J. C. (1998). Exopolymer production by intertidal epipelic diatoms. *Limnol. Oceanogr.* 43 1578–1591. 10.4319/lo.1998.43.7.1578

[B314] Smith D. J. Underwood G. J. C. (2000). The production of extracellular carbohydrates by estuarine benthic diatoms: the effects of growth phase and light and dark treatment. *J. Phycol.* 36 321–333. 10.1046/j.1529-8817.2000.99148.x

[B315] Sprachta S. Camoin G. Golubic S. Le Campion T. (2001). Microbialites in a modern lagoonal environment: nature and distribution, Tikehau atoll (French Polynesia). *Palaeogeogr. Palaeoclimatol. Palaeoecol.* 175 103–124. 10.1016/S0031-0182(01)00388-1

[B316] Steinberger R. E. Holden P. A. (2005). Extracellular DNA in single and multiple-species unsaturated biofilms. *Appl. Environ. Microbiol.* 71 5404–5410. 10.1128/AEM.71.9.5404-5410.2005 16151131 PMC1214645

[B317] Stewart P. S. (2002). Diffusion in biofilms. *J. Bacteriol.* 185 1485–1491. 10.1128/JB.185.5.1485-1491.2003 PMC148055 12591863

[B318] Stewart P. S. Franklin M. J. (2008). Physiological heterogeneity in biofilms. *Nat. Revs. Microbiol.* 6 199–210. 10.1038/nrmicro1838 18264116

[B319] Stoop J. M. H. Williamson J. D. Pharr D. M. (1996). Mannitol metabolism in plants: a method for coping with stress. *Trends Plant Sci.* 1 139–144. 10.1016/S1360-1385(96)80048-3

[B320] Suja L. D. Summers S. Gutierrez T. (2017). Role of EPS, dispersant and nutrients on the microbial response and MOS formation in the Subarctic Northeast Atlantic. *Front. Microbiol.* 8:676 10.3389/fmicb.2017.00676 PMC5399796 28484435

[B321] Sutherland I. W. (1999). “Polysaccharases in biofilms – sources – action – consequences,” in *Microbial Extracellular Polymeric Substances* eds Wingender J. Neu T. R. Flemming H.-C. (Berlin: Springer Press) 201–216. 10.1007/978-3-642-60147-7_11

[B322] Sutherland I. W. (2001). The biofilm matrix – an immobilized but dynamic microbial environment. *Trends Microbiol.* 9 222–227. 10.1016/S0966-842X(01)02012-1 11336839

[B323] Sutherland I. W. (2016). “EPS – a complex mixture,” in *The Perfect Slime – Microbial Extracellular Polymeric Substances* eds Flemming H.-C. Wingender J. Neu T. R. (London: IWA Publishers) 15–24.

[B324] Suzuki H. Daimon M. Awano T. Umekage S. Tanaka T. Kikuchi Y. (2009). Characterization of extracellular DNA production and flocculation of the marine photosynthetic bacterium *Rhodovulum sulfidophilum*. *Appl. Microbiol. Biotechnol.* 84 349–356. 10.1007/s00253-009-2031-7 19452150

[B325] Tang L. Schramm A. Neu T. R. Revsbech N. P. Meyer R. L. (2013). Extracellular DNA in adhesion and biofilm formation of four environmental isolates: a quantitative study. *FEMS Microbiol. Ecol.* 86 394–403. 10.1111/1574-6941.12168 23786537

[B326] Taylor M. W. Schupp P. J. Baillie H. J. Charlton T. S. de Nys R. Kjelleberg S. (2004). Evidence for acyl homoserine lactone signal production in bacteria associated with marine sponges. *Appl. Environ. Microbiol.* 70 4387–4389. 10.1128/AEM.70.7.4387-4389.2004 15240326 PMC444791

[B327] Teal J. M. Howarth R. W. (1984). Oil spill studies: a review of ecological effects. *Environ. Manag.* 8 27–44. 10.1007/BF01867871

[B328] Teske A. Ramsing N. B. Habicht K. Fukul M. Küver J. Jørgensen B. B. (1998). Sulfate-reducing bacteria and their activities in cyanobacterial mats of Solar lake (Sinai. Egypt). *Appl. Environ. Microbiol.* 64 2943–2951. 9687455 10.1128/aem.64.8.2943-2951.1998 PMC106797

[B329] Thavasi R. Banat I. M. (2014). “Biosurfactant and bioemulsifiers from marine sources-,” in *Biosurfactants: Research Trends and Applications* Chap. 5 eds Mulligan C. N. Sharma S. K. Hardback M. A. (Boca Raton, FL: CRC Press) 125–146. 10.1201/b16383-6

[B330] Thavasi R. Jayalakshmi S. Banat I. M. (2011). “Biosurfactant from marine bacterial isolates,” in *current Research Technology and Education Topics in Applied Microbiology and Microbial Biotechnology Book Series –* Vol. 2 ed. Mendez-Vilas A. (Badajoz: Formatex Research Center) 1367–1373.

[B331] Thingstad T. F. Hagström Å Rassoulzadegan F. (1997). Accumulation of degradable DOC in surface waters: is it caused by a malfunctioning microbial loop? *Limnol. Oceanogr.* 42 398–404. 10.4319/lo.1997.42.2.0398

[B332] Thingstad T. F. Li Zweifel U. Rassoulzadegan F. (1998). P limitation of heterotrophic bacteria and phytoplankton in the northwest Mediterranean. *Limnol. Oceanogr.* 43 88–94. 10.4319/lo.1998.43.1.0088

[B333] Thornton D. C. O. Fejes E. M. Dimarco S. F. Clancy K. M. (2007). Measurement of acid polysaccharides in marine and freshwater samples using alcian blue. *Limnol. Oceanogr. Method.* 5 73–87. 10.4319/lom.2007.5.73

[B334] Thornton D. C. O. Kopac S. M. Long R. A. (2010). Production and enzymatic hydrolysis of carbohydrates in intertidal sediments. *Aquat. Microb. Ecol.* 60 109–125. 10.3354/ame01403

[B335] Tice M. M. Lowe D. R. (2004). Photosynthetic microbial mats in the 3,416-Myr-old ocean. *Nature* 431 522–523. 10.1038/nature02888 15457255

[B336] Tielen P. Rosenau F. Wilhelm S. Jaeger K.-C. Flemming H.-C. Wingender J. (2013). Extracellular enzymes affect biofilm formation in mucoid *Pseudomonas aeruginosa*. *BMC Microbiol.* 159:221–228. 10.1099/mic.0.037036-0 20360178

[B337] Tiselius P. Kuylenstierna B. (1996). Growth and decline of a diatom spring bloom phytoplankton species composition, formation of marine snow and the role of heterotrophic dinoflagellates. *J. Plank. Res.* 18 133–155. 10.1093/plankt/18.2.133

[B338] Tolhurst T. J. Gust G. Paterson D. M. (2002). The influence of an extracellular polymeric substance (EPS) on cohesive sediment stability. *Proc. Marine Sci.* 5 409–425. 10.1016/S1568-2692(02)80030-4

[B339] Torti A. Lever M. A. Jorgensen B. B. (2015). Origin, dynamics and implications of extracellular DNA pools in marine sediments. *Mar. Genom.* 24 185–196. 10.1016/j.margen.2015.08.007 26452301

[B340] Tourney J. Ngwenya B. T. (2014). The role of bacterial extracellular polymeric substances in geomicrobiology. *Chem. Geol.* 386 115–132. 10.1128/AEM.06568-11

[B341] Tran C. Hadfield M. G. (2011). Larvae of *Pocillopora damicornis* (Anthozoa) settle and metamorphose in response to surface-biofilm bacteria. *Mar. Ecol. Prog. Ser.* 433 85–96. 10.3354/meps09192

[B342] Treguer P. Legendre L. Rivkin R. T. Ragueneau O. Dittert N. (2003). “Water column biogeochemistry below the euphotic zone,” in *Ocean Biogeochemistry: A Synthesis of the Joint Global Ocean Flux Study (JGOFS)* ed. Fasham J. R. M. (Berlin: Springer-Verlag) 145–156.

[B343] Tunnicliffe V. (1991). The biology of hydrothermal vents: ecology and evolution. *Oceanogr. Mar. Biol. Ann. Rev.* 29 319–408.

[B344] Tyree C. A. Hellion V. M. Alexandrova O. A. Allen J. O. (2007). Foam droplets generated from natural and artificial seawaters. *J. Geophys. Res.* 112:D12204 10.1029/2006jd007729

[B345] Ulrich M. (2009). *Bacterial Polysaccharides: Current Innovations and Future Trends.* Norwich: Caister Academic Press.

[B346] Unabia C. R. C. Hadfield M. G. (1999). Role of bacteria in larval settlement and metamorphosis of the polychaete *Hydroides elegans*. *Mar. Biol.* 133 55–64. 10.1007/s002270050442

[B347] Underwood G. J. C. Aslam S. N. Niemi A. Norman L. Meiners K. M. Laybourn-Parry J. (2013). Broad-scale predictability of carbohydrates and EPS in Antarctic and Arctic sea ice. *Proc. Natl. Acad. Sci. U.S.A.* 110 15734–15739. 10.1073/pnas.1302870110 24019487 PMC3785782

[B348] Underwood G. J. C. Boulcott M. Raines C. A. Waldron K. (2004). Environmental effects on exopolymer production by marine benthic diatoms – dynamics, changes in composition and pathways of production. *J. Phycol.* 40 293–304. 10.1111/j.1529-8817.2004.03076.x

[B349] Underwood G. J. C. Fietz S. Papadimitriou S. Thomas D. N. Dieckmann G. S. (2010). Distribution and composition of dissolved extracellular polymeric substances (EPS) in Antarctic sea ice. *Mar. Ecol. Prog. Ser.* 404 1–19. 10.3354/meps08557

[B350] Underwood G. J. C. Paterson D. M. (2003). The importance of extracellular carbohydrate production by marine epipelic diatoms. *Adv. Bot. Res.* 40 184–240. 10.1016/S0065-2296(05)40005-1

[B351] Underwood G. J. C. Paterson D. M. Parkes R. J. (1995). The measurement of microbial carbohydrate EPS from intertidal sediments. *Limnol. Oceanogr.* 40 1243–1253. 10.4319/lo.1995.40.7.1243

[B352] Valentine D. L. Fisher G. B. Bagby S. C. Nelson R. K. Reddy C. M. Sylva S. P. (2014). Fallout plume of submerged oil from Deepwater Horizon. *Proc. Natl. Acad. Sci. U.S.A* 111 15906–15911. 10.1073/pnas.1414873111 25349409 PMC4234598

[B353] van Gerven N. Klein R. D. Hultgren S. J. Remaut H. (2015). Bacterial amyloid formation: structural insights into curli biogenesis. *Trends Microbiol.* 23 693–706. 10.1016/j.tim.2015.07.010 26439293 PMC4636965

[B354] Varenyam A. Mukherjee A. Reddy M. S. (2010). Characterization of two urease-producing and calcifying *Bacillus* spp. Isolated from cement. *J. Microbiol. Biotechnol.* 20 1571–1576. 10.4014/jmb.1006.06032 21124064

[B355] Vasconcelos C. Warthmann R. McKenzie J. Visscher P. T. Bittermann A. G. van Lith Y. (2006). Lithifying microbial mats in lagoa vermelha, brazil: modern precambrian relics? *Sed. Geol.* 185 175–183. 10.1016/j.sedgeo.2005.12.022

[B356] Verdugo P. (1994). Polymer gel phase transition in condensation-decondensation of secretory products. *Adv. Polymer Sci.* 110 145–156. 10.1007/BFb0021131

[B357] Verdugo P. (2012). Marine microgels. *Ann. Rev. Marine Sci.* 4 375–400. 10.1146/annurev-marine-120709-142759 22457980

[B358] Verdugo P. Alldredge A. L. Azam F. Kirchman D. L. Passow U. Santschi P. (2004). The oceanic gel phase: a bridge in the DOM-POM continuum. *Mar. Chem.* 92 67–85. 10.1016/j.marchem.2004.06.017

[B359] Verdugo P. Santschi P. (2010). Polymer dynamics of DOC networks and gel formation in seawater. *Deep-Sea Res. II.* 57 1486–1493. 10.1016/j.dsr2.2010.03.002

[B360] Visscher P. T. Reid R. P. Bebout B. M. (2000). Microscale observations of sulfate reduction: evidence of microbial activity forming lithified micritic laminae in modern marine stromatolites. *Geology* 28 919–922. 10.1130/0091-7613(2000)28<919:MOOSRC>2.0.CO;2

[B361] Visscher P. T. Stolz J. F. (2005). Microbial mats as bioreactors: populations, processes, and products. *Palaeogeo. Palaeoclim. Palaeoecol.* 219 87–100. 10.1016/j.palaeo.2004.10.016

[B362] Vreeland R. H. Rosenzweig W. D. Powers D. W. (2000). Isolation of a 250 million-year-old halotolerant bacterium from a primary salt crystal. *Nature* 407 897–900. 10.1038/35038060 11057666

[B363] Wagner-Dobler I. Thiel V. Eberl L. Allgaier M. Bodor A. Meyer S. (2005). Discovery of complex mixtures of novel long-chain quorum sensing signals in free-living and host-associated marine alphaproteobacteria. *Chembiochem* 6 2195–2206. 10.1002/cbic.200500189 16283687

[B364] Waharte F. Steenkeste K. Briandet R. Fontaine-Aupart M. P. (2010). Diffusion measurements inside biofilms by image-based fluorescence recovery after photobleaching (FRAP) analysis with a commercial confocal laser scanning microscope. *Appl. Environ. Microbiol.* 76 5860–5869. 10.1128/AEM.00754-10 20639359 PMC2935062

[B365] Walker B. D. Beaupré S. R. Guilderson T. P. McCarthy M. D. Druffel E. R. M. (2016). Pacific carbon cycling constrained by organic matter size, age and composition relationships. *Nat. Geosci.* 9 888–891. 10.1038/ngeo2830

[B366] Waters C. M. Bassler B. L. (2005). Quorum sensing: cell-to-cell communication in bacteria. *Annu. Revs. Cell Dev. Biol.* 21 319–346. 10.1146/annurev.cellbio.21.012704.131001 16212498

[B367] Werlin R. Priester J. H. Mielke R. E. Kramer S. Jackson S. Stoimenov P. K. (2011). Biomagnification of cadmium selenide quantum dots in a simple experimental microbial food chain. *Nat. Nanotechnol.* 6 65–71. 10.1038/nnano.2010.251 21170041

[B368] Whalan S. Webster N. S. (2014). Sponge larval settlement cues: the role of microbial biofilms in a warming ocean. *Sci. Rep.* 4:4072 10.1038/srep04072 PMC3921630 24518965

[B369] Whitchurch C. B. Tolker-Nielsen T. Ragas P. C. Mattick J. S. (2002). Extracellular DNA required for bacterial biofilm formation. *Science* 295:1487 10.1126/science.295.5559.1487 11859186

[B370] Whitfield C. (2006). Biosynthesis and assembly of capsular polysaccharides in *Escherichia coli*. *Annu. Rev. Biochem.* 75 39–68. 10.1146/annurev.biochem.75.103004.142545 16756484

[B371] Widenfalk A. Lundqvist A. Goedkoop W. (2008). Sediment microbes and biofilms increase the bioavailability of chlorpyrifos in *Chironomus riparius* (Chironomidae, Diptera). *Ecotox. Environ. Saf.* 71 490–497. 10.1016/j.ecoenv.2007.10.028 18093655

[B372] Williams P. M. Druffel E. R. M. (1987). Radiocarbon in dissolved organic matter in the central North Pacific Ocean. *Nature* 330 246–248. 10.1038/330246a0

[B373] Wilson T. W. Ladino L. A. Alpert P. A. Breckels M. N. Brooks I. M. Browse J. (2015). A marine biogenic source of atmospheric ice-nucleating particles. *Nature* 525 234–238. 10.1038/nature14986 26354482

[B374] Wotton R. S. (2004). The ubiquity and many roles of EPS (EPS) in aquatic systems. *Oceanogr. Mar. Biol. Annu. Revs.* 42 57–94. 10.1201/9780203507810.ch3

[B375] Wurl O. (2009). *Practical Guidelines for the Analysis of Seawater.* Boca Raton, FL: CRC Press 10.1201/9781420073072

[B376] Wurl O. Holmes M. (2008). The gelatinous nature of the sea-surface microlayer. *Mar. Chem.* 110 89–97. 10.1016/j.marchem.2008.02.009

[B377] Wurl O. Miller L. Vagle S. (2011). Production and fate of transparent exopolymer particles in the ocean. *J. Geophys. Res.* 116:C00H13 10.1111/j.1462-2920.2012.02873.x

[B378] Yang C. Fang S. Chen D. Wang J. Liu F. Xia C. (2016). The possible role of bacterial signal molecules N-acyl homoserine lactones in the formation of diatom-biofilm (*Cylindrotheca* sp.). *Mar. Poll. Bull.* 107 118–124. 10.1016/j.marpolbul.2016.04.010 27090887

[B379] Yang M. Stipp S. L. S. Harding J. (2008). Biological control on calcite crystallization by polysaccharides. *Cryst. Growth Des.* 8 4066–4074. 10.1016/j.jsb.2015.03.007

[B380] Zhang G. Zhang F. Ding G. Li J. Guo X. Zhu J. (2012). Acyl homoserine lactone-based quorum sensing in a methanogenic archaeon. *ISME J.* 6 1336–1344. 10.1038/ismej.2011.203 22237544 PMC3379639

[B381] Zhang S. Xu C. Santschi P. H. (2008). Chemical composition and Thorium-234 (IV) binding of extracellular polymeric substances (EPS) by the marine diatom *Amphora* sp. *Mar. Chem.* 106 967–976.

[B382] Zhang W. Sun J. Ding W. Lin J. Tian R. Lu L. (2015). Extracellular matrix-associated proteins form an integral and dynamic system during *Pseudomonas aeruginosa* biofilm development. *Front. Cell. Infect. Microbiol.* 5:40 10.3389/fcimb.2015.00040 PMC4429628 26029669

[B383] Zheng G. Vad B. S. Dueholm M. S. Christiansen G. Nilsson M. Tolker-Nielsen T. (2015). Functional bacterial amyloid increases *Pseudomonas* biofilm hydrophobicity and stiffness. *Front. Microbiol.* 6:1099 10.3389/fmicb.2015.01099 PMC4595789 26500638

[B384] Ziervogel K. McKay L. Rhodes B. Osburn C. L. Dickson-Brown J. Arnosti C. (2012). Microbial activities and dissolved organic matter dynamics in oil- contaminated surface seawater from the Deepwater Horizon oil spill site. *PLoS ONE* 7:e34816 10.1371/journal.pone.0034816 PMC3324544 22509359

[B385] Zippel B. Neu T. R. (2011). Characterization of glycoconjugates of extracellular polymeric substances in Tufa-associated biofilms by using fluorescence lectin-binding analysis. *Appl. Environ. Microbiol.* 77 505–516. 10.1128/AEM.01660-10 21097578 PMC3020524

[B386] ZoBell C. E. Allen E. C. (1935). The significance of marine bacteria in the fouling of submerged surfaces. *J. Bact.* 29 239–251. 16559784 10.1128/jb.29.3.239-251.1935 PMC543592

[B387] Zohary T. Robarts R. D. (1998). Experimental study of microbial P limitation in the eastern Mediterranean. *Limnol. Oceanogr.* 43 387–395. 10.1007/s00248-015-0713-5

[B388] Zosim Z. Gutnick D. Rosenberg E. (1983). Uranium binding by emulsan and emulsanosols. *Biotechnol. Bioeng.* 25 1725–1735. 10.1002/bit.260250704 18551477

